# Moderate reductions in water and nitrogen inputs enhance lodging resistance of spring wheat under a novel drip irrigation pattern in Xinjiang

**DOI:** 10.3389/fpls.2026.1973875

**Published:** 2026-10-09

**Authors:** Yanhui Zhao, Dong Jiang, Haoyu Ding, Xiaoqi Ma, Min Song, Wanxu Zhang, Kai Xiao, Wenliang Wan

**Affiliations:** 1 State Key Laboratory of North China Crop Improvement and Regulation, Hebei Agricultural University, Baoding, China; 2 Key Laboratory of Oasis Eco-agriculture, Xinjiang Production and Construction Corps, Shihezi University, Shihezi, China; 3 Xinjiang Production and Construction Corps Agricultural Technology Extension Station, Urumqi, China

**Keywords:** lignin biosynthesis, lodging resistance, mechanical properties, spring wheat, water-nitrogen coupling

## Abstract

**Introduction:**

Drip irrigation improves water and fertilizer use efficiency in wheat. However, it may promote excessive stem elongation and shallow root development, increasing lodging risk. Although optimized water and nitrogen management can enhance lodging resistance in drip-irrigated wheat, the regulatory thresholds and underlying mechanisms remain unclear, particularly under the novel TR6S pattern.

**Methods:**

A two-year field experiment was conducted in 2024–2025 to systematically investigate the effects of water–nitrogen interactions on the lodging resistance of spring wheat under the TR6S pattern. Four irrigation levels were applied: 4500, 4200, 3900, and 3600 m^3^·ha^−1^, designated as W4500, W4200, W3900, and W3600, respectively. Three nitrogen application levels were applied: 300, 270, and 240 kg·ha^−1^, designated as N300, N270, and N240, respectively.

**Results:**

Wheat lodging rate exhibited a decreasing–increasing trend with reductions in both irrigation and nitrogen inputs. Compared with the traditional treatment (N300–W4500) (CK), moderate reductions in water–nitrogen inputs (N270–W4200) significantly increased the grain yield by 7.88–8.35% and reduced the lodging rate by 56.18–56.99%, achieving a favorable balance between yield (8.65–8.87 t·ha^−1^) and lodging rate (12.14–14.20%). Further reductions in water and nitrogen inputs decreased the lodging rate to some extent, and grain yield declined concurrently. The PLS-SEM model verified the reasons for the optimal lodging resistance performance of N270–W4200. Moderate nitrogen reduction enhanced the activities of key enzymes involved in lignin synthesis in basal stems, increased lignin content and stem firmness, and strengthened the mechanical properties against lodging, playing a major role in enhancing wheat lodging resistance. Moderate reduction in irrigation primarily increased root weight in the 30–60 cm soil layer, thereby enhancing root anchorage strength. However, as the irrigation volume did not efficiently regulate root growth in the middle soil layer (30–60 cm), it played only a secondary role in enhancing wheat lodging resistance.

**Discussion:**

This study quantified the optimal water–nitrogen input threshold for the TR6S pattern, with an irrigation volume ranging from 4203.55 to 4237.14 m^3^·ha^−1^ and the nitrogen application amount ranging from 264.49 to 283.16 kg·ha^−1^. These results provide a theoretical basis for promoting the TR6S pattern and technical references for studying crop lodging resistance in arid and semi-arid regions.

## 1 Introduction

Wheat (*Triticum aestivum* L.) is one of the three major food crops that provide a staple food source for approximately 35–40% of the global population (Shewry and Hey, 2015). It is also central to dietary cultures in many regions, including bread in Europe (Rezzouk et al., 2023), noodles in Asia (Wee and Jeyakumar Henry, 2019), and naan bread in the Middle East (Ahmed et al., 2021). However, freshwater scarcity severely restricts wheat production worldwide, particularly in arid and semi-arid regions (Chen et al., 2015). The Xinjiang Uygur Autonomous Region is a typical example, where annual evaporation can be up to 10 times greater than annual rainfall, imposing severe constraints on the agricultural water supply (Wan et al., 2022a). Therefore, efficient irrigation techniques are essential in Xinjiang. Since its introduction into wheat production in Xinjiang in 2008, drip irrigation has significantly improved water and fertilizer use efficiency (Wang et al., 2008).

However, as drip-irrigated wheat cultivation has expanded in Xinjiang, lodging risk has become increasingly serious (Li et al., 2017). Compared with flood irrigation, drip irrigation concentrates soil moisture and nutrients primarily in the upper soil layer (Lv et al., 2019). This leads to a shallow secondary root distribution, reduced root quantity, and weak root anchorage capacity in wheat (Wu et al., 2019; Ma et al., 2022). Although drip irrigation delivers water and nutrients more precisely to the root zone for rapid crop uptake, it often promotes excessive vegetative growth, basal internode elongation, and reduced stem mechanical strength (Li et al., 2017). When both roots and stems are weakened, the high temperatures and strong winds that often occur during the filling stage in Xinjiang can directly induce widespread lodging (Li et al., 2017; Wu et al., 2021). Lodging in drip-irrigated wheat can reduce yield by 20–30% and by more than 40% in severe cases (Wan et al., 2023). It reduces the mechanical harvesting efficiency and deteriorates grain quality (Zhang et al., 2024).

Currently, wheat lodging resistance is mainly managed through three measures: cultivar breeding, chemical regulation, and planting density. Nevertheless, these measures have several limitations when applied to drip-irrigated wheat production in Xinjiang. First, cultivar improvement, namely the breeding of density-tolerant cultivars, enhances lodging resistance by reducing plant height and optimizing population structure (Li et al., 2025a). However, density-tolerant cultivars must simultaneously possess complex traits, including lodging resistance, autonomous tiller regulation, compact plant type, and high photosynthetic efficiency. These traits are all regulated by polygenes, making targeted breeding extremely difficult (Shi et al., 2024). Second, chemical regulation acts by regulating the biosynthesis or signal transduction of endogenous plant hormones (e.g., gibberellin), thereby strengthening lodging resistance by reducing plant height and the height of the center of gravity (Lyu et al., 2021). However, Xinjiang experiences rapid warming in spring, resulting in a brief transition period from the tillering to the stem elongation stage in wheat (Wan et al., 2018). This imposes strict requirements on the timing, dosage, and application method of growth regulators, creating a high risk of inconsistent effects or excessive growth suppression. Third, reducing planting density lowers lodging risk by decreasing canopy closure, improving field ventilation and light transmittance, and curbing excessive basal internode elongation (Luo et al., 2019). However, high yield is the primary target for drip-irrigated wheat in Xinjiang, where relatively high sowing rates are widely adopted. Simply reducing planting density compromises yield potential and is therefore poorly accepted by producers (Li et al., 2025b). These constraints have made wheat lodging difficult to control effectively in Xinjiang in recent years, and lodging damage has become increasingly severe.

Our previous drip-irrigated wheat experiment showed that reduced water and nitrogen inputs had a significant anti-lodging effect. We selected TR6S (one drip tube served six wheat rows, with a ***t***ube-to-row ***r***atio of 1:*6* and a short inter-block spacing of 25 cm), a novel drip-irrigated wheat cultivation pattern, for efficient wheat production in Xinjiang (Wan et al., 2022a; **Supplementary Figure S1**). To identify the optimal water and nitrogen input levels for TR6S, our preliminary work examined how different water and nitrogen application rates affected wheat growth and development (Wan et al., 2022b). In this study, several plots with reduced water and nitrogen inputs demonstrated strong lodging resistance. Given the increasingly prominent lodging problem in drip-irrigated wheat in Xinjiang, this finding prompted further investigation. As one of the most critical agronomic practices in wheat cultivation, water and nitrogen management significantly regulates stem growth, root development, and lodging resistance (Li et al., 2022; Wang et al., 2022). Nitrogen application can increase plant height and aboveground biomass and promote yield formation. However, excessive nitrogen input increases the pectin content in stem cell walls, disrupts the balance between cellulose and hemicellulose, reduces stem mechanical strength, and consequently increases the stem lodging risk (Luo et al., 2019; Li et al., 2022). Water supply has similar effects. Irrigation promotes wheat growth and development, softens the soil, reduces root anchorage capacity, and increases root lodging risk (Pinera-Chavez et al., 2016; Dreccer et al., 2020). In contrast, moderate soil nitrogen and water conditions can regulate the activities of enzymes related to endogenous substances in roots and stems, thereby enhancing the overall plant lodging resistance (Chen et al., 2018; Wang et al., 2022).

Drip irrigation technology provides an important basis for the precise regulation of water and fertilizer. However, the physiological mechanisms by which water–nitrogen coupling regulates wheat lodging resistance under the novel TR6S planting pattern remain underexplored. Therefore, using the established TR6S pattern, this study set different water and nitrogen gradients to systematically investigate their coupling effects on wheat lodging resistance and yield. The primary objectives of this study were to (1) determine whether moderate reductions in water and nitrogen inputs enhance wheat lodging resistance under the TR6S drip irrigation pattern, (2) identify the optimal water–nitrogen treatment for the synergistic improvement of grain yield and lodging resistance, and (3) clarify the physiological mechanisms responsible for enhanced lodging resistance under optimal water–nitrogen treatment. These results can support sustainable drip-irrigated wheat production in Xinjiang and provide theoretical and technical support for the high-yield, lodging-resistant cultivation of drip-irrigated wheat in other arid regions.

## 2 Materials and methods

### 2.1 Experimental design

Field experiments were conducted during the 2024 and 2025 spring wheat growing seasons at the Wheat Experiment Farm of Shihezi University (44°21′N, 86°04′E; altitude: 450 m). Sowing was performed on April 6 and April 8, followed by harvesting on July 16 and July 15 in 2024 and 2025, respectively. The experimental soil was sandy loam, and the physical and chemical properties of the 0–90 cm soil layer are listed in **Table 1**. Across the two growing seasons, the total solar radiation was 2188 and 2294 MJ·m^−2^, and the accumulated temperature ≥10 °C was 2068.6 °C and 2273.4 °C, respectively. The precipitation during the wheat growing season at the experimental field was 67.29 mm and 35.15 mm in 2024 and 2025, respectively. Daily meteorological data, including maximum and minimum temperatures, precipitation, and monthly average wind speed, were recorded during the experimental period (**Figure 1**).

**Table 1:** Basic physical and chemical properties of soil at 0–90 cm depth before sowing.

Soil- depth (cm)	Soil- texture	pH	Field water- capacity (%)	Total- porosity (%)	Soil organic- matter (g kg^−1^)	Bulk- density (g kg^−1^)	Available-N (mg kg^−1^)	Olsen -P (mg kg^−1^)	Available- potassium (mg kg^−1^)
0–30	loam	7.62 ± 0.28	26.82 ± 1.14	45.43 ± 1.65	11.5 ± 0.51	1.24 ± 0.03	42.3 ± 1.72	13.9 ± 1.16	295 ± 4.62
30–60	loam	7.56 ± 0.22	25.71 ± 1.11	45.24 ± 1.34	10.7 ± 0.46	1.28 ± 0.03	41.6 ± 1.44	13.3 ± 1.18	286 ± 3.91
60–90	loam	7.66 ± 0.24	25.22 ± 0.85	44.71 ± 1.26	10.5 ± 0.37	1.32 ± 0.02	40.9 ± 1.35	12.6 ± 1.09	283 ± 3.85

**Figure 1:**
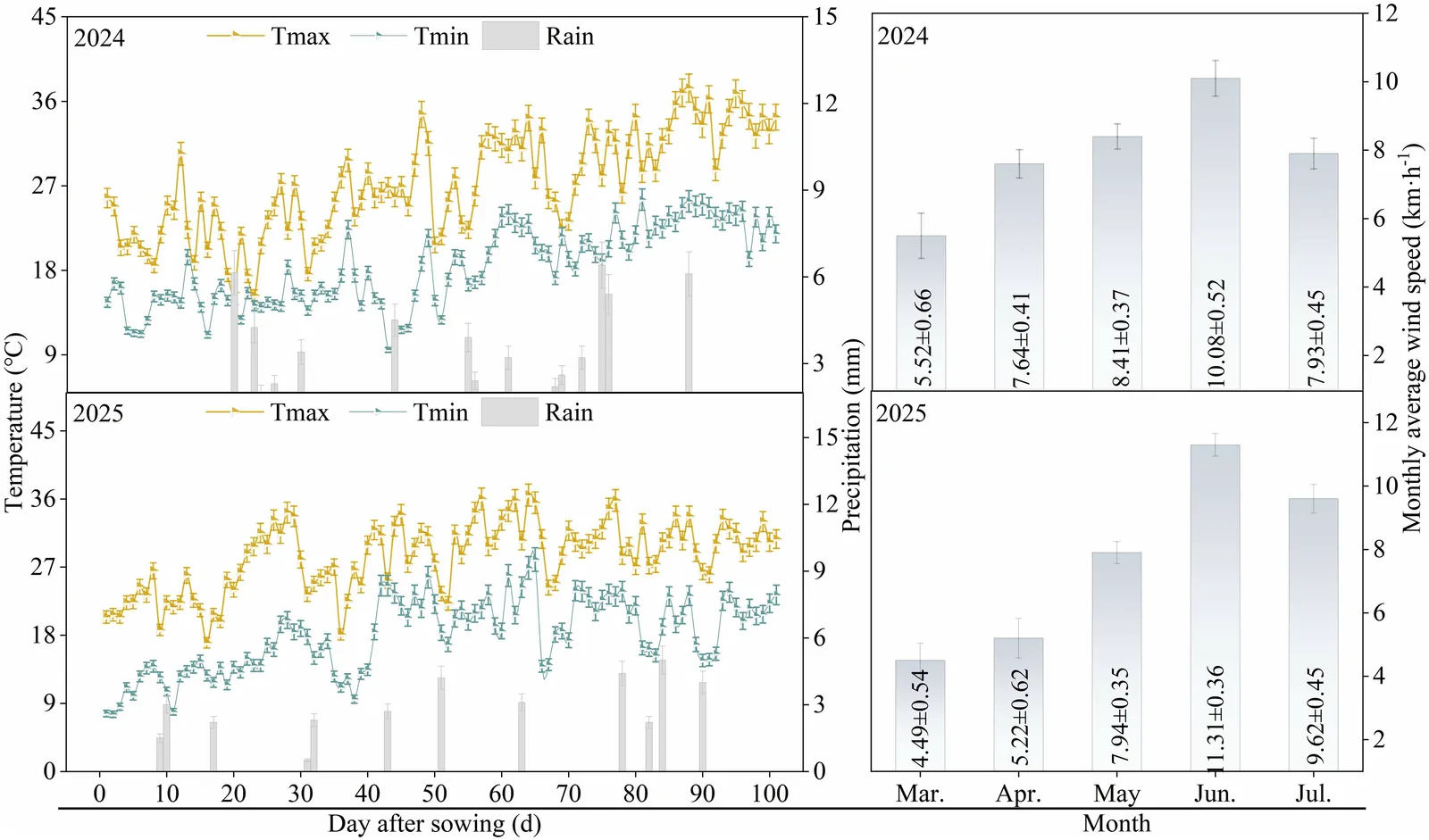
Daily meteorological data during the experimental period of spring wheat. Tmax, Tmin, and Rain represent the daily maximum temperature, daily minimum temperature, and daily precipitation, respectively, throughout the growth period of the drip-irrigated spring wheat. Data were collected from the National meteorological information centre.

The local traditional water and nitrogen application rates, 4500 m^3^·ha^−1^ irrigation and 300 kg·ha^−1^ nitrogen (Lv et al., 2019), were used as the control (CK), with reduced water and nitrogen treatments established accordingly. Four whole-season irrigation levels were applied, including 4500 (conventional full irrigation), 4200 (moderate deficit irrigation), 3900 (medium deficit irrigation), and 3600 m^3^·ha^−1^ (severe deficit irrigation), denoted as W4500, W4200, W3900, and W3600, respectively. Three whole-season nitrogen application levels were applied: 300, 270, and 240 kg·ha^−1^, denoted as N300, N270, and N240, respectively. The nitrogen gradient was designed based on the measured soil nitrogen conditions of the experimental field (**Table 1**) and local production practices (Lv et al., 2019). N300 represented the conventional nitrogen application rate used by local farmers and served as the control (Wan et al., 2022a). N270 was close to the economically optimal nitrogen application threshold of 280 kg·ha^−1^ for this variety (Yang, 2020). It also addresses the need for further reductions in the use of chemical fertilizers. N240 represented a greater nitrogen reduction. This treatment aimed to define the lower limit of nitrogen application that could maintain grain yield and improve lodging resistance under the TR6S pattern. Irrigation water was mainly obtained from underground wells, and nitrogen was supplied primarily as urea (CH_4_N_2_O; 46% N). The experiment followed a two-factor randomized block design with 12 treatments (four irrigation levels × three nitrogen application rates), three field replicates per treatment, and 36 plots in total. Each plot measured 45 m^2^ (5 m × 9 m). Fertilizer tanks and water meters were installed separately in each plot to control the water and fertilizer application. To prevent both surface and subsurface lateral water and nutrient interference between adjacent plots, a 1.2 m wide buffer zone was established between all neighboring plots. Plastic film barriers were installed vertically to a depth of 1.5 m along the plot boundaries. Sowing was performed using a specialized TR6S-adapted seeder (JB/T 6274.1-2013, 2BFX-12, China), which enabled simultaneous seed sowing and drip tube laying (**Supplementary Figure S2**). Plant density was maintained consistently across treatments at 550 × 10^4^ plants·ha^−1^ per plot. The locally cultivated spring wheat cultivar Xinchun 44 (XC44) was used in this study. Based on the experience from high-yield fields, phosphate fertilizer (superphosphate; P_2_O_5_ content ≥12%) and potassium fertilizer (potassium sulfate; K_2_O content ≥50%) were applied entirely as basal fertilizers during soil preparation before sowing. Each fertilizer was applied once at 105 kg·ha^−1^ (Lv et al., 2019; Wan et al., 2022a). Irrigation and fertilization strategies at each wheat growth stage followed the methods described by Lv et al. (2019) (**Table 2**).

**Table 2:** Irrigation and nitrogen application ratios for spring wheat during different growth stages in 2024 and 2025.

Growth stage	Pre-sowing	Two-leaf-stage (8 d)	Jointing-stage (32 d)	Booting stage (48 d)	Anthesis (62 d)	Early milk stage (74 d)	Late milk stage (78 d)
Irrigation ratio	—	20%	20%	20%	15%	15%	10%
Nitrogen ratio	20%	12%	32%	16%	12%	8%	—

Values in parentheses correspond to the phenological phases of wheat as defined by Zadoks et al. (1974).

### 2.2 Sampling and measurements

#### 2.2.1 Lodging rate and grain yield

Wheat lodging in Xinjiang mainly occurs during the filling stage (Li et al., 2017). Therefore, the lodging rate was measured for each treatment at this stage. Because the drip irrigation was spatially non-uniform, wheat growth varied among rows (in the TR6S planting pattern, the first row (R1), second row (R2), and third row (R3) were located at different distances from the drip tube). To avoid the influence of inter-row differences, the lodging rate was measured in a 1.6 m^2^ sampling area that included all wheat rows, with 0.8 m width between adjacent drip tubes and 2 m row length. The total number of plants and lodged plants in the area were counted. A plant was considered lodged when the inclination angle between the wheat stem and the ground was ≤45° (Keller et al., 1999). The lodging rate for each treatment was calculated using Equation 1:

(1) $$\text{Lodging rate }\left(\%\right)\;=\;\left(\text{the number of lodged plants of }1.6\;{\text{m}}^{2}\text{ area}/\text{the number of all plants in }1.6\;{\text{m}}^{2}\text{ area}\right)\;\times \;100$$

Grain yield was determined at maturity. Nine samples were harvested from three experimental plots, with each sample covering an area of 1.6 m^2^. After threshing, the grains were weighed. Grain yield per unit area (g·m^−2^) was calculated as the grain weight divided by the sampling area and then converted to yield per hectare (t·ha^−1^).

#### 2.2.2 Stalk bending strength, stalk rind penetration strength, and single plant anchorage strength

Stem mechanical resistance to lodging in wheat is commonly assessed using stalk bending strength (SBS) and stalk rind penetration strength (SRPS) (Peng et al., 2014). Following Peng et al. (2014), the SBS and SRPS of the second basal internode were measured using a stalk strength tester (TP-YYD-1; Hangzhou Top Instrument Co., Ltd., China). At the grain-filling stage, 30 wheat plants per treatment, with 10 plants from each row, were selected. For SBS, the second basal internode was placed horizontally in the grooves of two supporting columns spaced 5 cm apart, and pressure was applied perpendicular to the stem using the SBS probe until the internode broke. The maximum force at breakage was recorded as SBS and expressed in Newtons (N). For SRPS, another set of 30 intact second basal internodes with leaf sheaths were collected from each treatment and placed in the same supporting configuration. The SRPS probe was applied perpendicular to the stem surface until the epidermis was punctured, and the maximum penetration force was recorded as SRPS and expressed in Newtons (N). Root mechanical resistance to lodging was evaluated using single-plant anchorage strength (SPAS) (Wu et al., 2019). At the grain-filling stage, 15 wheat plants per treatment, with five plants from each row, were selected. The basal stems of the five plants from each row were bundled with a nylon rope, the other end of which was attached to the hook of a stalk strength tester mounted on a steel triangular stand above the plants. A uniform vertical upward force was applied until the root–soil complex was completely pulled out, and the maximum peak force during extraction was recorded as SPAS and expressed in Newtons (N). For each treatment, SBS, SRPS, and SPAS were averaged across all measured rows.

#### 2.2.3 Stalk pole substantial degree, stalk lignin content, and key enzyme activities in lignin biosynthesis

At the grain-filling stage, 30 wheat plants per treatment, including 10 plants from each row, were randomly selected from each experimental plot to determine the stalk pole substantial degree (SPSD) according to Luo et al. (2019). The second basal internode, including the sheath, was measured using a vernier caliper, heated at 105 °C for 30 min to deactivate enzymes, and dried at 85 °C to a constant weight. SPSD was calculated as the internode dry weight divided by the internode length (g·cm^−1^). Concurrently, 15 samples were selected from each plot for leaf sheath removal from the second basal internode (i.e., 15 plants from each row). These samples were immediately frozen in liquid nitrogen for 30 min and stored at −80 °C for the determination of stalk lignin content (SLC) and the activities of key enzymes involved in lignin biosynthesis. SLC was determined as described by Wang et al. (2015).

Phenylalanine ammonia-lyase (PAL) activity was analyzed based on the method of Assis et al. (2001). Briefly, 0.5 g of stalk tissue was ground in 0.05 mol·L^−1^ borate-boric acid buffer (pH 8.8) supplemented with mercaptoethanol, ethylenediaminetetraacetic acid, and polyvinylpyrrolidone. After centrifugation (10,000 rpm for 15 min at 4 °C), the supernatant was collected. The reaction mixture containing the enzyme extract and L-phenylalanine was incubated at 37 °C for 30 min and then terminated by boiling. Absorbance was measured at 290 nm against a blank lacking the substrate, and PAL activity was calculated based on the hourly change in A_290_.

Tyrosine ammonia-lyase (TAL) activity was measured according to the method described by Kofalvi and Nassuth (1995), using the enzyme extract described above. The reaction mixture containing tyrosine was incubated at 37 °C for 30 min and then terminated by boiling. Absorbance was measured at 315 nm, and enzyme activity was calculated from the hourly change in A_315_.

4-Coumarate-CoA ligase (4CL) activity was assessed as described by Knobloch and Hahlbrock (1975). A 0.5 g stalk sample was homogenized in ice-cold Tris-HCl buffer and centrifuged (10,000 rpm for 15 min). The supernatant was mixed with a reaction solution containing coumaric acid, ATP, CoA-SH, and MgSO_4_·7H_2_O and incubated at 40 °C for 10 min. Absorbance was measured at 333 nm, and enzyme activity was calculated from the hourly change in A_333_.

Cinnamyl alcohol dehydrogenase (CAD) activity was measured as described by Morrison et al. (1994). A 0.4 g stalk sample was ground in phosphate buffer and centrifuged (10,000 rpm for 20 min at 4 °C). The supernatant was incubated with the reaction substrates at 37 °C for 30 min. Absorbance was measured at 340 nm, and enzyme activity was calculated based on the hourly change in A_340_.

#### 2.2.4 Root length and root weight

Root morphological characteristics were determined at the grain-filling stage as described by Li et al. (2020). Root–soil complexes were collected across rows R1, R2, and R3 from the 0–30, 30–60, and 60–90 cm soil layers using a handheld soil drill with an inner diameter of 5.5 cm and a height of 30 cm. The samples were washed in a stainless steel sieve with a 0.25 mm^2^ mesh to remove adhered soil particles, followed by separation of roots using tweezers and transfer into a water-filled transparent glass container. High-resolution black-and-white TIF images were obtained using a root scanner (Epson V500, USA) and analyzed using WinRHIZO image analysis software (Canada). Total root length (TRL) was calculated for each soil layer, with treatment values expressed as the average across all measured rows. The root samples were subsequently dried at 80 °C to a constant weight and weighed using an analytical balance with an accuracy of 0.0001 g. Total root weight (TRW) was calculated for each soil layer, with the treatment values expressed as the average across all measured rows.

### 2.3 Statistical analysis

A two-way analysis of variance was conducted using IBM SPSS 26.0 to evaluate the effects of nitrogen application rate, irrigation amount, and their interaction on key indicators, including plant lodging rate. Multiple comparisons were performed using the least significant difference test (*P* < 0.05). Partial least squares structural equation modeling (PLS-SEM) was performed using SmartPLS 4.0 to quantify and verify the relationships among measured indicators. All charts were generated using Origin 2024.

## 3 Results

### 3.1 Moderate reductions in water and nitrogen inputs can enhance lodging resistance in drip-irrigated spring wheat, increasing and stabilizing yield

As shown in **Table 3**, at a fixed nitrogen application rate, the reduced irrigation amount produced a U-shaped response in wheat lodging rate. Treatments were ranked as W3600 > W4500 > W3900 > W4200, with significant differences in most cases. The grain yield showed an opposite inverted U-shaped response, with the treatments ranked as W4200 > W4500 > W3900 > W3600, again with significant differences in most cases. These responses were most pronounced under N270. Compared with the control (N300–W4500), W4200 significantly reduced lodging rate by 56.18–56.99% and increased grain yield by 7.88–8.35%. Moreover, W3900 significantly reduced the lodging rate by 47.54–50.18% but significantly decreased the yield by 2.78–3.60%. In contrast, W3600 reduced the lodging rate by only 4.10–7.08%, with significance detected in 2024 but not in 2025, and caused a significant yield reduction of 17.34–17.87%.

**Table 3:** Lodging rate and grain yield of spring wheat under different water and nitrogen treatments in the 2024 and 2025 seasons.

Year		2024				2025			
Treatment		Lodging rate		Grain yield		Lodging rate		Grain yield	
		%	DT_CK_ (%)	t·ha^−1^	DT_CK_ (%)	%	DT_CK_ (%)	t·ha^−1^	DT_CK_ (%)
N300	W4500	32.40 ± 0.85^bc^		8.02 ± 0.04^c^		28.23 ± 1.09^bc^		8.19 ± 0.05^b^	
	W4200	27.28 ± 1.06^e^	-15.80	8.26 ± 0.02^b^	2.98	23.95 ± 1.34^e^	-15.16	8.84 ± 0.03^a^	7.97
	W3900	29.17 ± 1.59^d^	-9.97	7.73 ± 0.05^d^	-3.62	25.51 ± 1.20^de^	-9.63	7.97 ± 0.02^c^	-2.70
	W3600	32.91 ± 1.32^b^	1.57	6.81 ± 0.08^f^	-15.15	28.80 ± 1.17^bc^	2.01	7.03 ± 0.11^f^	-14.13
N270	W4500	15.71 ± 1.22^fg^	-51.51	8.06 ± 0.02^c^	0.55	15.93 ± 1.45^f^	-43.56	8.24 ± 0.04^b^	0.59
	W4200	14.20 ± 1.48^g^	-56.18	8.65 ± 0.08^a^	7.88	12.14 ± 1.44^g^	-56.99	8.87 ± 0.09^a^	8.35
	W3900	16.14 ± 1.51^f^	-50.18	7.73 ± 0.07^d^	-3.60	14.81 ± 1.15^f^	-47.54	7.96 ± 0.07^c^	-2.78
	W3600	30.11 ± 1.71^d^	-7.08	6.51 ± 0.06^g^	-18.87	27.07 ± 1.25^cd^	-4.10	6.77 ± 0.04^g^	-17.34
N240	W4500	30.78 ± 1.59^cd^	-5.00	7.27 ± 0.05^e^	-9.32	27.20 ± 1.04^cd^	-3.64	7.54 ± 0.01^d^	-7.90
	W4200	30.72 ± 1.21^cd^	-5.20	7.35 ± 0.03^e^	-8.29	27.12 ± 1.22^cd^	-3.93	7.62 ± 0.01^d^	-6.90
	W3900	33.12 ± 1.38^b^	2.22	6.92 ± 0.04^f^	-13.70	29.87 ± 1.43^ab^	5.81	7.19 ± 0.04^e^	-12.22
	W3600	35.94 ± 1.24^a^	10.93	5.79 ± 0.05^h^	-27.79	31.72 ± 1.16^a^	12.38	6.05 ± 0.02^h^	-26.15
*F_N_*		283.67**		302.89**		246.45**		336.87**	
*F_W_*		62.03**		489.47**		55.34**		464.54**	
*F_N × W_*		14.51**		7.88**		16.22**		13.73**	

N300, N270, and N240 represent nitrogen application rates of 300, 270, and 240 kg·ha^−1^, respectively. W4500, W4200, W3900, and W3600 represent irrigation amounts of 4500, 4200, 3900, and 3600 m^3^·ha^−1^, respectively. The treatment combining 300 kg·ha^−1^ nitrogen and 4500 m^3^·ha^−1^ irrigation (N300–W4500) was set as the control (CK). DT_CK_ denotes the percentage change in plant lodging rate and grain yield for each treatment relative to CK. Different lowercase letters within the same column indicate statistically significant differences among treatments at *P* < 0.05. *F_N_*, *F_W_*, and *F_N × W_* represent the *F-values* for the effects of nitrogen application, irrigation amount, and their interaction, respectively. ** indicates a significant difference at *P <* 0.01.

At a fixed irrigation amount, reducing the nitrogen application rate produced a similar U-shaped response in wheat lodging rate, with the treatments ranked as N240 > N300 > N270 and significant differences in most cases. In contrast, the grain yield exhibited an inverted U-shaped response, with the order N270 > N300 > N240 and significant differences in most cases. These responses were most pronounced under W4200. Reducing the nitrogen application rate to N270 significantly decreased the lodging rate by 56.18–56.99% and increased the yield by 7.88–8.35% compared with CK (N300–W4500). Further reduction to N240 decreased the lodging rate by only 3.93–5.20%, with no significant difference from the control, but it significantly reduced the yield by 6.90–8.29%.

Across both experimental years, N270–W4200 demonstrated the best combined performance and was identified as the optimal water–nitrogen management strategy in this experiment for balancing lodging resistance with a high yield. Furthermore, the response surface analysis defined the optimal input ranges under the TR6S pattern as 264.49–283.16 kg·ha^−1^ for the nitrogen application and 4203.55–4237.14 m^3^·ha^−1^ for irrigation (**Figure 2**). The metrics of nitrogen application rate, irrigation amount, and their interaction all significantly affected the lodging rate and grain yield in drip-irrigated wheat (*P* < 0.01), and the main effects of nitrogen and irrigation were stronger than those of their interaction. The nitrogen application rate had a substantially stronger effect on lodging rate (*F_N_* = 246.45–283.67) than the irrigation amount (*F_W_* = 55.34–62.03). The irrigation amount exerted a stronger effect on grain yield (*F_W_* = 464.54–489.47) than the nitrogen application rate (*F_N_* = 302.89–336.87).

**Figure 2:**
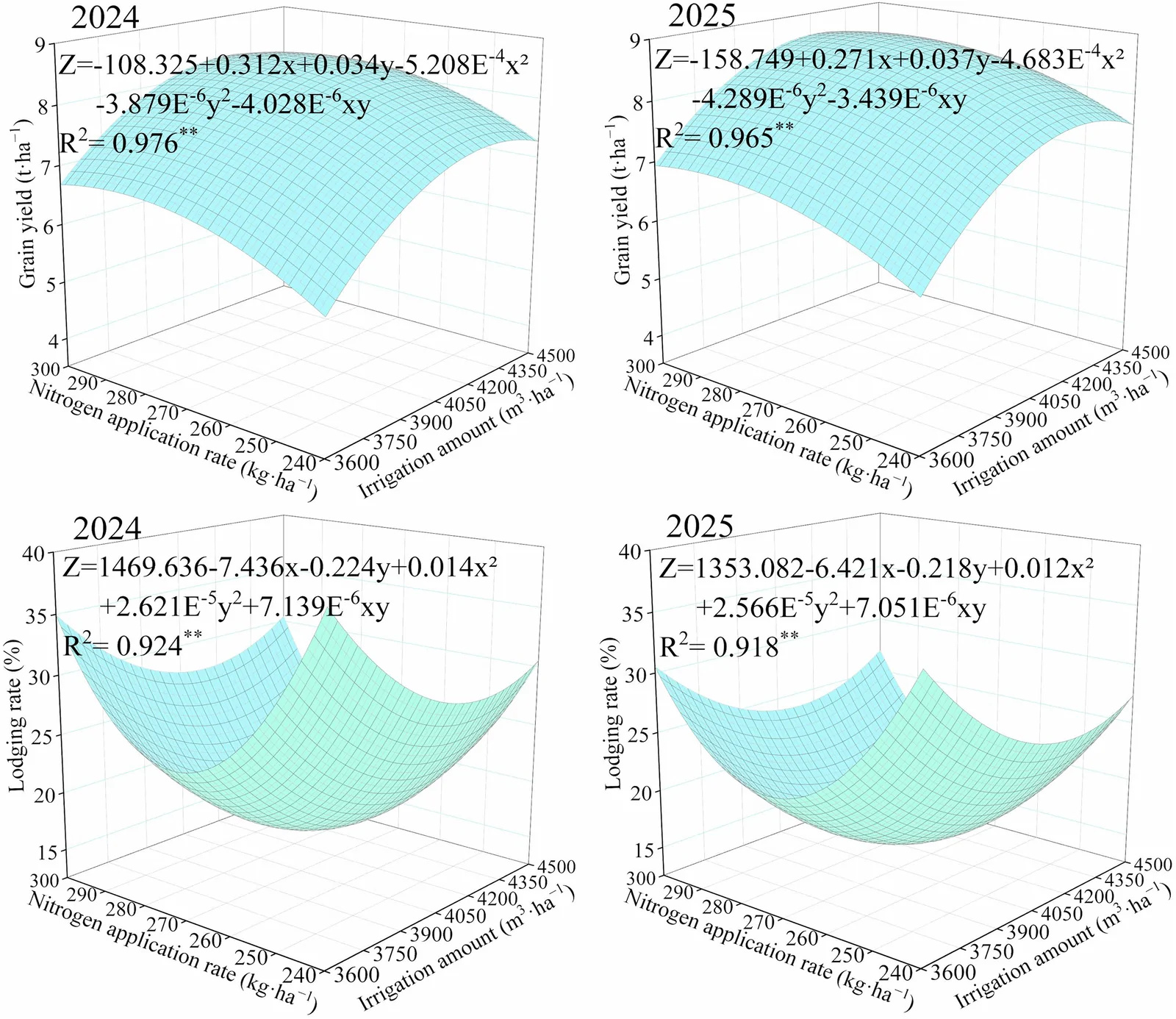
Response surface analysis of lodging rate and grain yield of spring wheat under different water and nitrogen treatments.

### 3.2 Stem mechanical properties dominate lodging resistance, whereas root anchorage strength plays a secondary role

At a fixed nitrogen application rate, reductions in irrigation amount produced an inverted U-shaped response in SBS, SRPS, and SPAS, closely corresponding to the variation in lodging rate (**Figure 3**; **Table 3**). The response was most pronounced under N270. Compared with the CK (N300–W4500), W4200 significantly increased SBS, SRPS, and SPAS by 21.86–22.59%, 19.31–25.65%, and 7.32–7.80%, respectively. W3900 produced smaller but still significant increases of 5.39–8.21%, 5.36–8.31%, and 4.69–5.86%, respectively. In contrast, further reduction to W3600 significantly decreased SBS, SRPS, and SPAS by 3.91–4.69%, 3.12–5.16%, and 3.72–4.06%, respectively. These results indicate that compared with the traditional irrigation amount in Xinjiang (W4500), W4200 most effectively enhanced both stem mechanical properties (SBS and SRPS) and root anchorage strength (SPAS).

**Figure 3:**
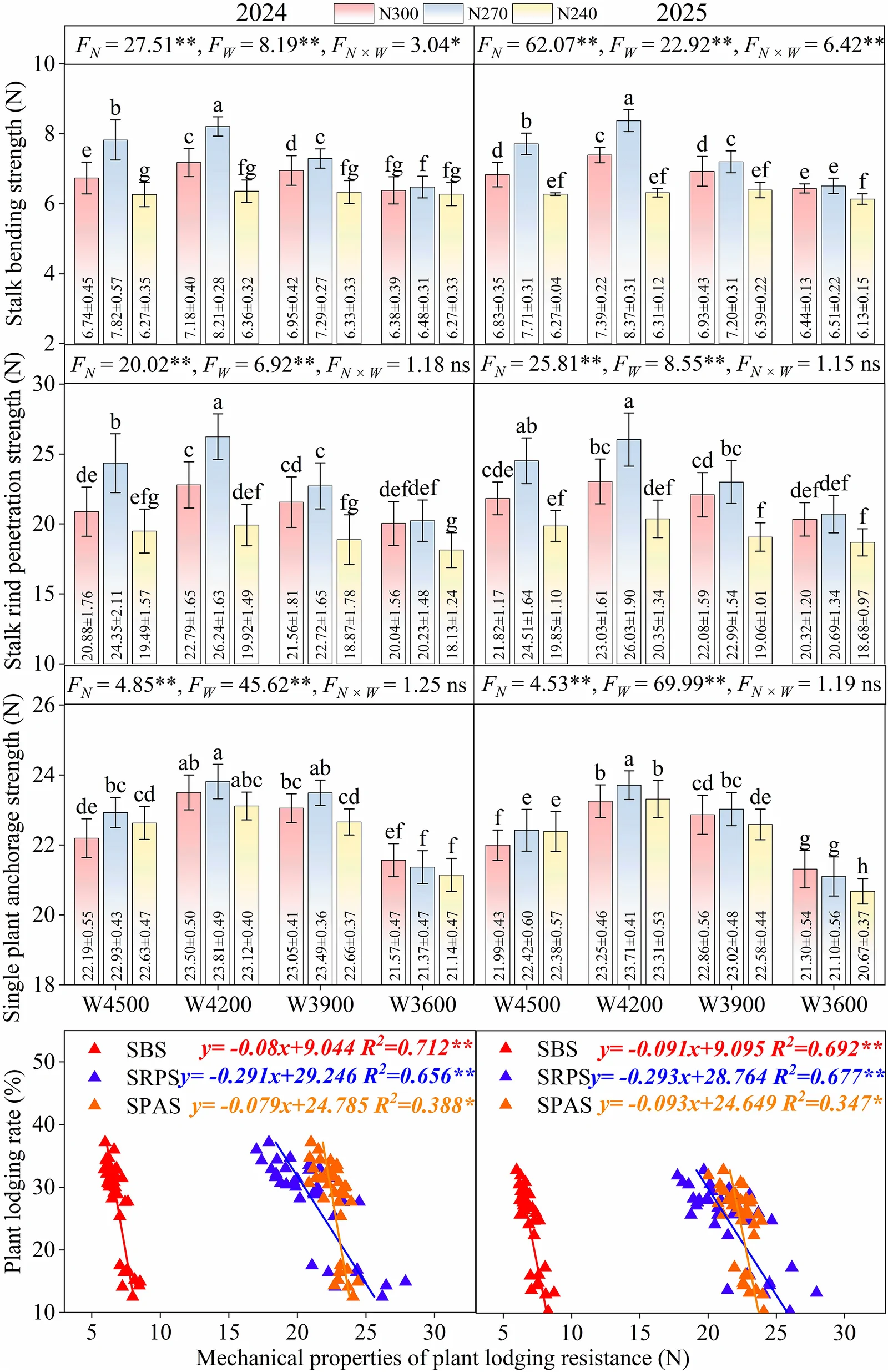
Variations in basal stem mechanical properties and single-plant anchorage strength and their associations with lodging rate. N300, N270, and N240 represent nitrogen application rates of 300, 270, and 240 kg·ha^−1^, respectively. W4500, W4200, W3900, and W3600 represent irrigation amounts of 4500, 4200, 3900, and 3600 m^3^·ha^−1^, respectively. The treatment combining 300 kg·ha^−1^ nitrogen and 4500 m^3^·ha^−1^ irrigation (N300–W4500) was set as the control (CK). Different lowercase letters indicate statistically significant differences between treatments at *P* < 0.05. *F_N_*, *F_W_*, and *F_N × W_* represent the *F-values* for effects of nitrogen application rate, irrigation amount, and their interaction, respectively. ** and * indicate significant differences at *P* < 0.01 and *P* < 0.05 levels, respectively; ns indicates no significant difference.

At a consistent irrigation amount, reducing the nitrogen application rate induced a similar inverted U-shaped response in SBS, SRPS, and SPAS, corresponding closely to the variation in lodging rate (**Figure 3**; **Table 3**). This response was most pronounced under W4200. Reducing the nitrogen application rate to N270 significantly increased SBS, SRPS, and SPAS by 21.86–22.59%, 19.31–25.65%, and 7.32–7.80%, respectively, compared with CK (N300–W4500). Further reduction to N240 significantly decreased SBS and SRPS by 5.69–7.56% and 4.58–6.72%, respectively, whereas SPAS still increased slightly by 4.17–5.99%, with significant differences. Thus, compared with the traditional nitrogen application rate in Xinjiang (N300), N270 provided the best overall improvement in stem mechanical properties (SBS and SRPS) and root anchorage strength (SPAS).

Correlation analysis confirmed that the lodging rate decreased almost linearly as anti-lodging mechanical properties (SBS, SRPS, and SPAS) increased. The correlations of lodging rate with stem mechanical properties (SBS and SRPS) (*R*^2^ = 0.656–0.712) were stronger than those with root anchorage strength (SPAS) (*R*^2^ = 0.347–0.388), indicating that stem strengthening contributed more strongly to lodging resistance than root anchorage. The main effect analysis supported this pattern. The nitrogen application rate exerted a stronger effect on stem mechanical indicators (SBS and SRPS) (*F_N_* = 20.02–62.07) than the irrigation amount (*F_W_* = 6.92–22.92). The irrigation amount exerted a stronger effect on SPAS (*F_W_* = 45.62–69.99) than the nitrogen application rate (*F_N_* = 4.53–4.85) (**Figure 3**).

### 3.3 Nitrogen predominantly regulates stem mechanical properties through increased substantial degree and lignin content

At the same nitrogen application rate, reducing the irrigation amount produced an inverted U-shaped response in SPSD and SLC (**Figure 4**). The response was most pronounced under N270. Compared with CK (N300–W4500), W4200 significantly increased SPSD and SLC by 21.39–24.57% and 11.11–14.36%, respectively. W3900 produced smaller but still significant increases of 3.48–5.06% and 8.32–9.01%, respectively. In contrast, further reduction to W3600 significantly reduced SPSD and SLC by 3.09–6.47% and 7.27–7.35%, respectively. Compared with the traditional irrigation amount in Xinjiang (W4500), W4200 provided the greatest improvement in lignin content and a substantial degree of basal wheat stems.

**Figure 4:**
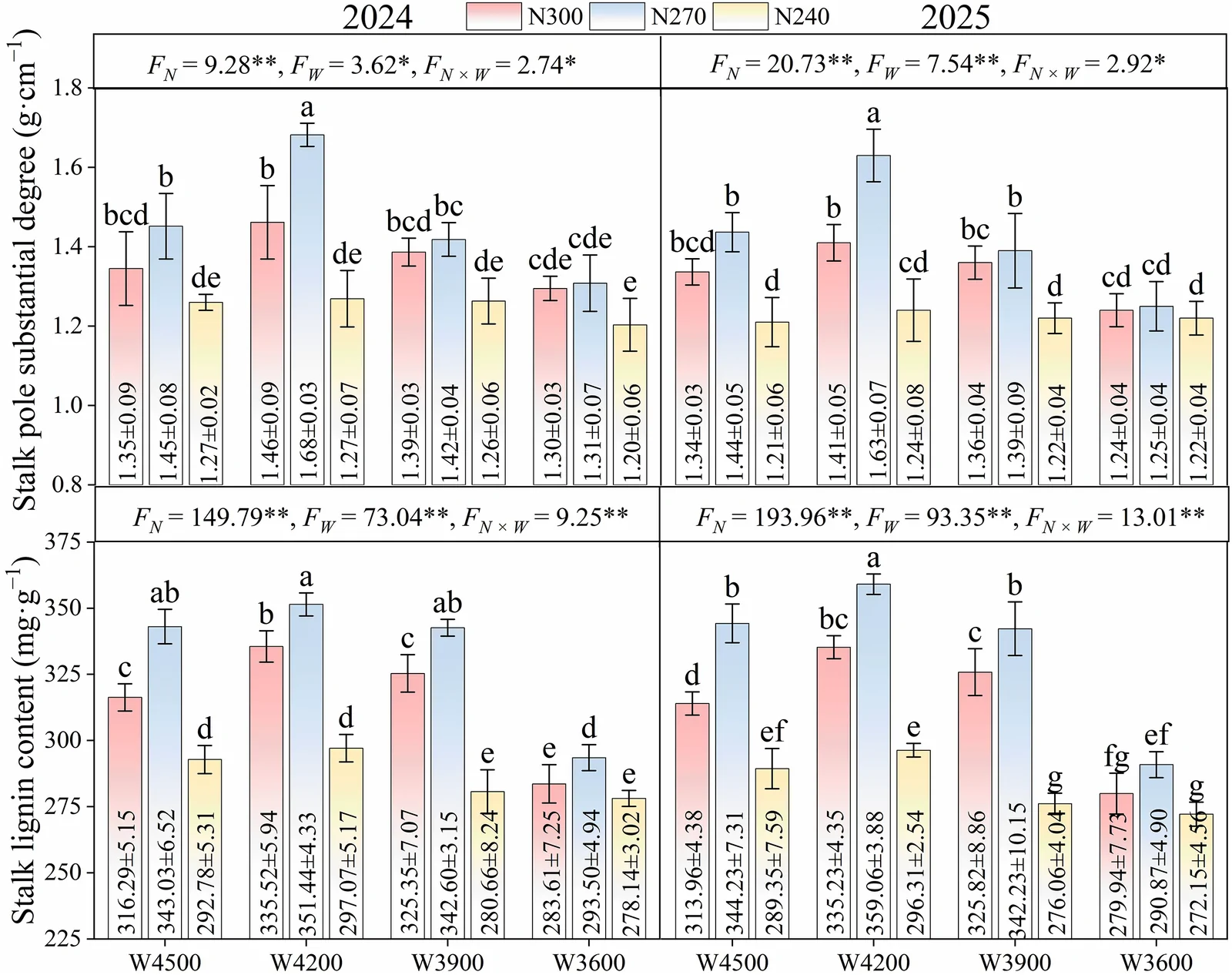
Effects of irrigation and nitrogen levels on stalk pole substantial degree and stalk lignin content of the second internode.

At a fixed irrigation amount, reducing the nitrogen application rate induced a similar inverted U-shaped response in SPSD and SLC (**Figure 4**), with the strongest response under W4200. Reducing the nitrogen application rate to N270 significantly increased SPSD and SLC by 21.39–24.57% and 11.11–14.36%, respectively, compared with CK (N300–W4500). Further reduction to N240 significantly decreased SPSD and SLC by 6.27–7.21% and 5.62–6.08%, respectively. Compared with the traditional nitrogen application rate in Xinjiang (N300), N270 was the most effective treatment for increasing the substantial degree and lignin content of basal wheat stems.

Nitrogen application rate, irrigation amount, and their interaction significantly affected both SPSD and SLC (*P* < 0.05), and the main effects of nitrogen and irrigation were stronger than their interaction effect (**Figure 4**). The nitrogen application rate exerted a stronger regulatory effect on SPSD (*F_N_* = 9.28–20.73) and SLC (*F_N_* = 149.79–193.96) than the irrigation amount (SPSD: *F_W_* = 3.62–7.54; SLC: *F_W_* = 73.04–93.95).

### 3.4 Moderate nitrogen reduction enhances the activities of key enzymes involved in lignin biosynthesis

At a fixed nitrogen application rate, reducing the irrigation amount produced an inverted U-shaped response in the activities of PAL, TAL, 4CL, and CAD (**Figure 5**), with the strongest response under N270. Compared with CK (N300–W4500), W4200 significantly increased PAL, TAL, 4CL, and CAD activities by 11.21–12.04%, 16.07–20.52%, 16.58–21.72%, and 13.19–18.60%, respectively. W3900 produced smaller increases of 2.62–3.50%, 5.05–6.58%, 4.60–6.36%, and 1.09–1.78%, respectively, whereas these differences were not significant in most cases. In contrast, further reduction to W3600 significantly decreased PAL, TAL, 4CL, and CAD activities by 7.32–8.03%, 6.38–10.13%, 5.71–6.81%, and 3.52–9.84%, respectively. Thus, W4200 was the optimal irrigation level for enhancing the activities of these four lignin-biosynthesis enzymes, consistent with the response of lignin content to irrigation amount (**Figures 4**, **5**).

**Figure 5:**
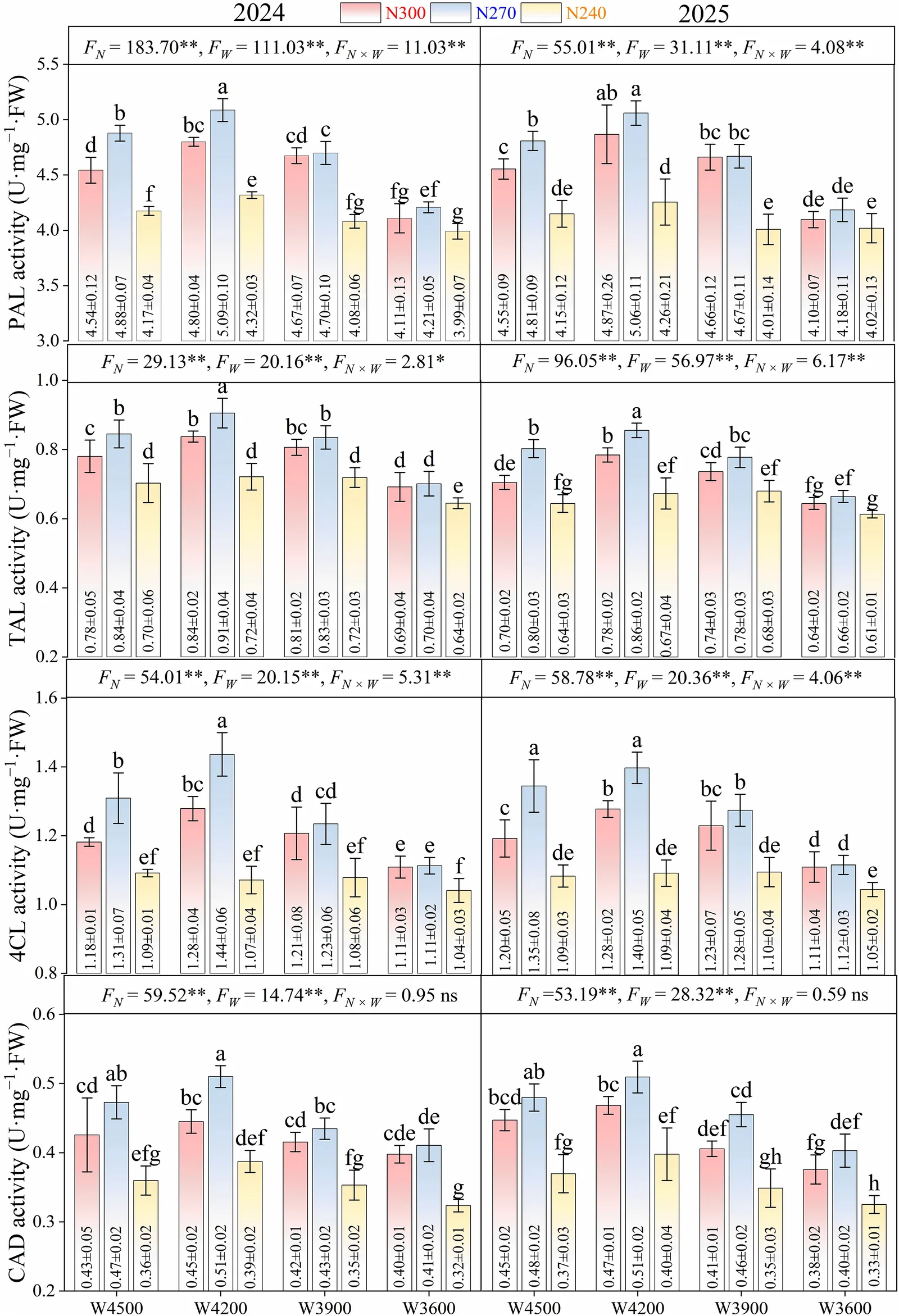
Effects of irrigation and nitrogen levels on the activities of key lignin biosynthetic enzymes in the second internode.

At the same irrigation amount, reducing the nitrogen application rate induced a similar inverted U-shaped response in PAL, TAL, 4CL, and CAD activities (**Figure 5**), with the strongest response under W4200. Compared with CK (N300–W4500), reducing the nitrogen application rate to N270 significantly increased PAL, TAL, 4CL, and CAD activities by 11.21–12.04%, 16.07–20.52%, 16.58–21.72%, and 13.19–18.60%, respectively. Further reduction to N240 significantly decreased the activities of these four enzymes by 4.87–6.47%, 5.19–7.56%, 8.81–9.21%, and 9.92–11.63%, respectively. Therefore, N270 was the optimal nitrogen application rate for enhancing lignin-biosynthesis enzyme activities, consistent with the response of lignin content to nitrogen application rate.

Analysis of variance demonstrated that both the nitrogen application rate and irrigation amount significantly affected the activities of key enzymes involved in lignin biosynthesis (*P* < 0.01). The main-effect analysis identified the nitrogen application rate as a stronger regulator of PAL, TAL, 4CL, and CAD activities (*F_N_* = 29.13–183.70) than irrigation amount (*F_W_* = 14.74–111.03).

### 3.5 Moderate irrigation reduction enhances root anchorage through improved root growth in the 30–60 cm soil layer

The root responses to irrigation reduction varied significantly with soil depth (**Figure 6**). At a fixed nitrogen application rate, decreasing the irrigation amount continuously reduced the TRL and TRW in the shallow soil layer (0–30 cm). In the 30–60 cm middle soil layer, TRL and TRW exhibited an inverted U-shaped response and peaked under W4200. In contrast, both traits increased continuously in the 60–90 cm deep soil layer as the irrigation amount decreased. A similar depth-dependent response was observed for nitrogen reduction with a fixed irrigation amount. Decreasing the nitrogen application rate continuously reduced TRL and TRW in the 0–30 cm layer, produced peak values under N270 in the 30–60 cm layer, and continuously increased both traits in the 60–90 cm layer.

**Figure 6:**
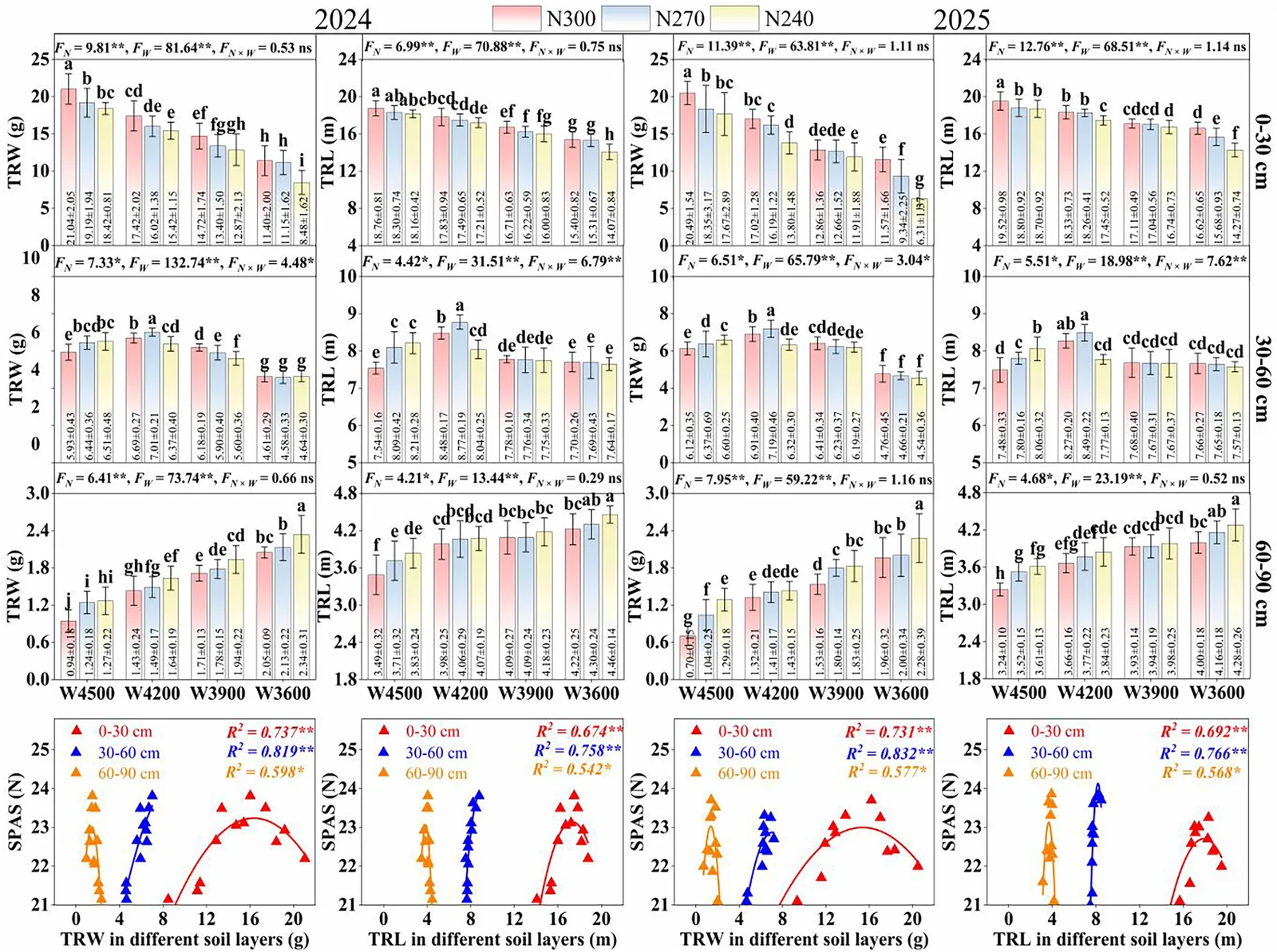
Vertical distribution patterns of total root weight (TRW) and total root length (TRL) in response to water and nitrogen treatments in drip-irrigated spring wheat.

Correlation analysis identified the 30–60 cm soil layer as the key layer controlling root anchorage strength. The correlations of TRL and TRW with SPAS were strongest in the 30–60 cm layer (*R*^2^ = 0.758–0.832), exceeding those in the 0–30 cm (*R*^2^ = 0.674–0.737) and 60–90 cm layers (*R*^2^ = 0.542–0.598). Within the 30–60 cm layer, TRW was more strongly correlated with SPAS (*R*^2^ = 0.819–0.832) than with TRL (*R*^2^ = 0.758–0.766). This indicates that root biomass in this layer is the core determinant of root anchorage strength and contributes more to lodging resistance than root length. For the 30–60 cm layer, further analysis was performed to compare the effects of irrigation, nitrogen, and their interaction on root growth traits (TRL and TRW) in drip-irrigated wheat. Irrigation had a stronger effect (*F_W_* = 18.98–132.74) than nitrogen (*F_N_* = 4.22–7.33) or their interaction (*F_N × W_* = 3.04–7.62).

### 3.6 PLS-SEM supports the proposed mechanisms underlying lodging resistance

The PLS-SEM model supported the proposed regulatory pathways, with an overall goodness-of-fit (GoF) index of 0.776, indicating an excellent model fit (**Figure 7**). The model identified two major pathways by which reduced water and nitrogen application regulated the lodging rate of drip-irrigated wheat. In the nitrogen-regulated pathway, the reduced nitrogen application rate had a direct positive effect on the activities of key enzymes involved in stalk lignin biosynthesis (PAL, TAL, 4CL, and CAD) (path coefficient (*β*) = 0.668, *P* < 0.001). It also had a direct positive effect on root growth status in the 0–30 cm soil layer (TRW and TRL) (*β* = 0.280, *P* < 0.001). It had a direct but non-significant positive effect on TRW and TRL in the 30–60 cm layer (*β* = 0.026, *P* > 0.001). It directly and negatively affected root growth in the 60–90 cm layer (*β* = −0.286, *P* < 0.001). The path coefficients indicated that the nitrogen application rate more strongly regulated the lignin-biosynthesis enzyme activities than root growth status. These enzyme activities directly increased SLC (*β* = 0.950, *P* < 0.001). Increased SLC directly promoted SPSD (*β* = 0.728, *P* < 0.001) and indirectly improved stem lodging resistance mechanical properties (SBS and SRPS) (*β* = 0.649, *P* < 0.001). SPSD strengthened these mechanical properties, which ultimately reduced the lodging rate through a direct negative effect (*β* = −0.575, *P* < 0.001).

**Figure 7:**
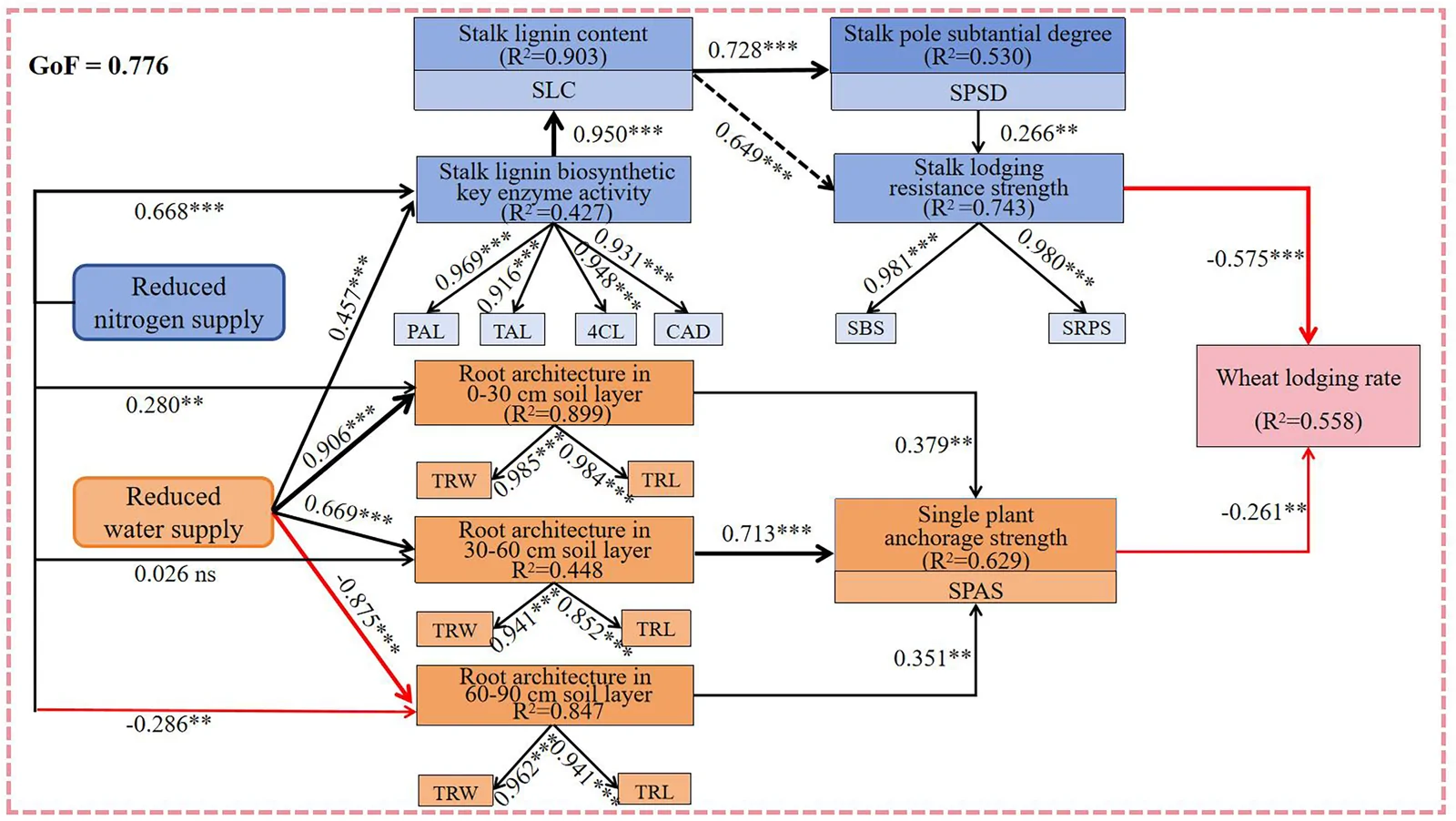
Partial least squares structural equation modeling (PLS-SEM) illustrating the dual-pathway mechanisms by which water and nitrogen reduction regulates lodging resistance in spring wheat under the TR6S pattern. The GoF = 0.776 in the upper left corner represents the overall goodness-of-fit index of the model. The blue and orange rounded rectangles represent exogenous variables (reduced nitrogen supply and reduced water supply), and the rectangular boxes represent latent variables and observed indicators. The latent variables included lignin biosynthesis enzyme activities, stalk lignin content (SLC), stalk pole substantial degree (SPSD), stem mechanical properties associated with lodging resistance, root architecture in different soil layers, single-plant anchorage strength (SPAS), and wheat lodging rate. The enzyme activities measured included phenylalanine ammonia-lyase (PAL), tyrosine ammonia-lyase (TAL), 4-coumarate-CoA ligase (4CL), and cinnamyl alcohol dehydrogenase (CAD). Stem mechanical properties included stalk bending strength (SBS) and stalk rind penetration strength (SRPS). Root architecture was represented by total root weight (TRW) and total root length (TRL). Arrows pointing from latent variables to subordinate small boxes (observed indicators) represent reflective measurement paths, with annotated values indicating the standardized factor loadings that reflect the contribution of observed indicators to latent variables and measurement reliability. The *R*^2^ values within the latent variable rectangles indicate the proportion of variance explained by the upstream paths in the model. The black solid arrows represent positive effect paths, the red solid arrows represent negative effect paths, and the dashed arrows represent secondary or indirect regulatory paths. Values adjacent to the arrows are standardized path coefficients (*β*), with larger absolute values indicating stronger regulatory effects of the antecedent variables on the outcome variables. Significance levels are denoted as follows: *** indicates *P* < 0.001, ** indicates *P* < 0.01, and ns indicates non-significant (*P* > 0.05). The blue and orange modules represent the “nitrogen reduction-driven stem lignification and mechanical strengthening” pathway, and the orange module represents the “water reduction-driven root system reconstruction in the plow layer and anchorage strength enhancement” pathway.

In the irrigation-regulated pathway, the reduced irrigation amount directly increased root growth status (TRW and TRL) in the 0–60 cm soil layer (*β* = 0.669, *P* < 0.001). It also directly increased the activities of key enzymes involved in stalk lignin biosynthesis (PAL, TAL, 4CL, and CAD) (*β* = 0.457, *P* < 0.001). However, it directly decreased root growth in the 60–90 cm layer (*β* = −0.875, *P* < 0.001). The path coefficients indicated that the irrigation amount more strongly regulated root growth status than lignin-biosynthesis enzyme activities. Its effect was stronger in the 0–30 cm layer (*β* = 0.906, *P* < 0.001) than in the 30–60 cm layer (*β* = 0.669, *P* < 0.001). However, root growth in the 30–60 cm layer exerted the strongest direct positive effect on SPAS (*β* = 0.713, *P* < 0.001). This exceeded the effects of the 0–30 cm layer (*β* = 0.379, *P* < 0.01) and 60–90 cm layers (*β* = 0.351, *P* < 0.01). SPAS directly and negatively affected the lodging rate (*β* = −0.261, *P* < 0.01), thereby improving wheat lodging resistance.

## 4 Discussion

Drip irrigation can achieve an irrigation efficiency of approximately 80%, far exceeding that of surface flood irrigation (approximately 35%) and sprinkler irrigation (approximately 75%) in open fields (Chen et al., 2015). In 2008, Xinjiang developed the TR4 drip-irrigation configuration, in which one drip tube supplies four wheat rows, with a tube-row ratio of 4, row spacing of 15 cm, and 60 cm between adjacent drip tubes. Compared with flood irrigation, TR4 can substantially save irrigation water and increase wheat yield (Chen et al., 2015). However, as drip-irrigated wheat cultivation expands, lodging has become increasingly prominent (Li et al., 2017; Wan et al., 2023). Over the past three years, the annual lodged area of drip-irrigated wheat has reached approximately 30% or more, making lodging-resistance regulation a major research focus in Xinjiang wheat cultivation (Wan et al., 2023). During the optimization of drip-irrigated planting patterns, we observed pronounced lodging resistance in wheat grown under reduced irrigation and nitrogen application. Because TR4 requires a high drip-tube investment, we previously developed a wide–narrow row pattern with a larger tube-row ratio, TR6S, by increasing the number of wheat rows supplied by each drip tube. In TR6S, one drip tube supplies six wheat rows with 10 cm row spacing, 80 cm between adjacent drip tubes, and 25 cm between border rows (Wan et al., 2022a). Compared with TR4, TR6S reduced the drip tube costs by 12.32% and increased the economic benefits by 3.44–5.35%, while maintaining yields essentially equivalent to those under TR4 (Wan et al., 2022a). Given its promotion potential, we examined water–nitrogen coupling under TR6S to clarify suitable water and fertilizer management strategies. Notably, several reduced-input plots showed pronounced lodging resistance, along with an increase in lodging severity in Xinjiang drip-irrigated wheat. This finding prompted the present study on reduced water and nitrogen application under the novel TR6S planting pattern.

The two-year fixed-site field experiment confirmed that under the TR6S pattern in Xinjiang, moderate reductions in water and nitrogen inputs enhanced wheat lodging resistance while improving both yield stability and grain yield. This is consistent with previous studies showing that appropriate reductions in water and nitrogen inputs can optimize crop architecture and strengthen lodging resistance (Li et al., 2022; Wang et al., 2022). Excessive water and nitrogen inputs usually disturb the carbon–nitrogen metabolic balance in wheat and weaken its lodging resistance (Chen et al., 2018; Li et al., 2022). In this experiment, lodging rate and grain yield showed significant quadratic responses to water and nitrogen input levels under both fixed nitrogen and fixed irrigation conditions. As the irrigation amount and nitrogen application rate decreased, the lodging rate decreased initially and then increased, whereas the grain yield increased initially and then decreased, consistent with previous findings. Moreover, the nitrogen application rate affected lodging rate more strongly than irrigation amount, whereas the irrigation amount exerted a stronger effect on grain yield than nitrogen application rate. In Xinjiang drip-irrigated wheat production, the lodging resistance is mainly regulated by nitrogen input, whereas grain yield is mainly controlled by irrigation input. This pattern differs from the water–fertilizer management experience in humid wheat-growing regions (Pinera-Chavez et al., 2016) and may reflect a region-specific strategy for extremely arid environments such as Xinjiang, where annual evapotranspiration exceeds annual precipitation by more than 10-fold. Therefore, nitrogen management should be prioritized for lodging control, and irrigation management should be prioritized for yield improvement. Among all treatments, N270–W4200 provided the best balance between lodging resistance and high yield, reducing the lodging rate by 56.18–56.99% and increasing the grain yield by 7.88–8.35% compared with the local traditional N300–W4500 combination. Further reductions in irrigation and nitrogen could lower lodging risk, but only with substantial yield loss. Using response surface fitting, we quantified the optimal water and nitrogen levels for drip-irrigated wheat under the TR6S pattern in Xinjiang (**Figure 2**). The results indicated that nitrogen application rates of 264.49–283.16 kg·ha^−1^ combined with irrigation amounts of 4203.55–4237.14 m^3^·ha^−1^ could synergistically improve wheat lodging resistance and grain yield. Investigations have shown that the average grain yield of spring wheat in the Xinjiang Production and Construction Corps reaches approximately 7500 kg·ha^−1^. The present experiment adopted a series of measures, including the TR6S planting pattern and water and nitrogen regulation. These measures can substantially increase grain yield relative to the benchmark and improve economic benefits. This further verifies the great potential for cost savings and efficiency improvement in this experiment.

The improved lodging resistance was primarily driven by enhanced stem mechanical properties, with root anchorage strength as a secondary contributor. Currently, the indicators for evaluating wheat lodging resistance mainly include morphological and mechanical indicators (Khan et al., 2019; Feng et al., 2024). In our previous study, path analysis examined morphological indicators associated with lodging resistance in XC44 under the TR6S pattern, including plant height, center-of-gravity height, and flag leaf angle. These indicators mainly regulate lodging risk indirectly through canopy photosynthesis and stem substance accumulation, whereas mechanical indicators play the main direct role in lodging performance (Wan et al., 2023). As this study was conducted using the same variety and planting pattern, we only used the relevant mechanical indicators of lodging resistance to evaluate wheat lodging resistance. In fact, whether stem lodging or root lodging dominates in drip-irrigated wheat has long been debated in Xinjiang (Xiong et al., 2012; Li et al., 2017; Wan et al., 2023). Under the traditional equal-row-spacing TR4 pattern, wheat is generally considered more prone to root lodging (Xiong et al., 2012). In the present study, the stem mechanical traits associated with lodging resistance (SBS and SRPS) closely paralleled the variation in lodging rate and demonstrated higher correlations with lodging rate than SPAS (**Figure 2**; **Table 3**). Although SPAS varied with lodging rate, the correlation was weaker, indicating that stem lodging was the dominant lodging type under TR6S. This differed from the lodging pattern inferred for TR4 and may be associated with the improved ventilation and light interception of the wide–narrow row arrangement. These conditions may improve soil aeration, support stronger root growth, and enhance root anchorage capacity. In this study, we did not measure morphological traits, including the height of the plant’s center of gravity, basal internode length and diameter, and culm wall thickness, to evaluate stem lodging. Our previous research showed that basal stem mechanical properties predominantly and directly influence lodging in drip-irrigated wheat, whereas these morphological traits have only indirect effects (Wan et al., 2023). Further analysis revealed that compared with the irrigation amount, the nitrogen application rate exerted a considerably greater effect on stem mechanical indicators (SBS and SRPS). Compared with the nitrogen application rate, the irrigation amount had a substantially greater effect on plant anchorage strength (SPAS). This indicates that wheat lodging resistance was achieved primarily through the regulation of stem lodging resistance mechanical properties by the nitrogen application rate and the regulation of root anchorage strength by irrigation. Therefore, under wide–narrow row drip irrigation patterns, such as TR6S, particular attention should be paid to stem lodging, and reasonable nitrogen management strategies could serve as important regulatory measures. In contrast, under equal-row-spacing drip irrigation patterns, such as TR4, attention should be paid to root lodging, and reasonable irrigation strategies can serve as important regulatory measures. This indicates that planting patterns exerted a considerable influence on the lodging types of drip-irrigated wheat, which should be emphasized in future research.

The improvement in stem mechanical properties can be mainly explained by the increase in SPSD and SLC. The nitrogen application rate is the dominant regulator of endogenous structural substance accumulation in stems. SPSD reflects the degree of filling of the stem parenchyma tissue, whereas SLC, as a major cell wall structural component, is essential for stem rigidity and toughness. Overall, they determine the basal stem mechanical strength (Peng et al., 2014; Luo et al., 2019). In the present study, both SPSD and SLC showed quadratic responses to water and nitrogen gradients, with moderate reductions increasing both traits, whereas excessive or insufficient inputs reduced them (**Figure 3**). This supports previous evidence that wheat stem development has strict water–fertilizer thresholds. High-water and high-nitrogen regimes inhibit lignification and reduce the stem’s substantial degree, which can affect stem mechanical strength (Feng et al., 2024). Conversely, appropriate water–nitrogen inputs can coordinate carbon–nitrogen metabolism, increase stem filling and lignin content, and improve stem mechanics (Li et al., 2022). Based on the relative chlorophyll content measured using SPAD readings of drip-irrigated wheat in Xinjiang, we previously constructed a nitrogen stage recommendation model. This suggests that nitrogen application during early growth should be reduced by approximately 60% for drip-irrigated wheat. Reducing total nitrogen application by 10–25 kg·ha^-1^ also better matches wheat nitrogen nutritional status (Wan et al., 2022c). This was highly consistent with the nitrogen range quantified, 264.49–283.16 kg·ha^-1^ (**Figure 2**), indicating that appropriate nitrogen nutrition could support stem structural substance accumulation and mechanical lodging resistance. It also suggests that the nitrogen nutrition index may be useful for lodging evaluation. Because wheat in arid regions is highly water-sensitive (Yang, 2020) and drip irrigation is spatially non-uniform (Chen et al., 2015), determining the optimal water requirement is complex. Our previous δ^13^C-based analysis demonstrated that wheat water use efficiency under wide–narrow row patterns, such as TR6S, was significantly higher than that under the traditional equal-row-spacing TR4 pattern (Wan et al., 2022d). This implies a lower water demand under TR6S and explains why reduced irrigation could also optimize stem structural substances. Nevertheless, the nitrogen application rate regulated the stem substantial degree, especially lignin accumulation, more strongly than irrigation amount did (**Figure 4**). Mechanistically, nitrogen can directly contribute to the formation of lignin biosynthesis precursors and regulate key enzymes in carbon metabolism. This serves as a decisive nutritional factor in cell wall lignification. Even slight changes in the nitrogen supply can markedly alter the accumulation of stem structural substances (Khan et al., 2019). In contrast, irrigation amount mainly affects stem substance accumulation indirectly through plant water metabolism and photosynthetic rate, with a more moderate regulatory intensity (Zhang et al., 2024, 2026). This explains why nitrogen dominated the improvement in the physicochemical properties of stems in this study.

The moderate reduction in the nitrogen application rate optimized lignin biosynthesis enzyme activities, thereby promoting lignin accumulation in the basal stem internodes. The phenylpropanoid pathway is the core route for plant lignin biosynthesis. PAL and TAL, as initial rate-limiting enzymes, catalyze the production of lignin biosynthesis precursors (Shi et al., 2013; Vanholme et al., 2019). Moreover, 4CL and CAD are key downstream enzymes that regulate lignin monomer synthesis, reduction, and polymerization (Ma, 2010; Nguyen et al., 2016). Together, these four enzymes determine the lignin accumulation rate and total deposition in plant stems, a regulatory pattern widely confirmed in lodging-resistance studies of graminaceous crops (Chen et al., 2018; Khan et al., 2019; Li et al., 2023). High-water and high-nitrogen regimes can suppress key phenylpropanoid enzymes, impede precursor conversion, reduce monomer polymerization efficiency, restrict lignin accumulation, and weaken stem mechanical strength (Li et al., 2022). Moderate water–nitrogen optimization can alleviate high-nitrogen metabolic inhibition, activate lignin biosynthesis enzymes, facilitate upstream and downstream metabolic flux, and promote lignin monomer synthesis and cell wall deposition (Li et al., 2022). However, severe water or nitrogen deficiency suppresses plant metabolism, reduces enzyme activity, and restricts lignin biosynthesis, thereby limiting the formation of lodging-resistant plant architectures (Li et al., 2022). In the present study, PAL, TAL, 4CL, and CAD activities were highly synchronized with lignin content and showed clear quadratic responses (**Figures 4**, **5**), consistent with previous findings. Furthermore, the main-effect analysis confirmed the central regulatory role of nitrogen application rate. Compared with irrigation amount, the nitrogen application rate had a dominant effect on the activities of these four enzymes. This is consistent with the role of nitrogen as a fundamental substrate for enzyme protein synthesis and a regulator of gene expression and enzyme activation. In contrast, irrigation can indirectly affect enzyme activity through plant water physiology and photosynthetic substrate supply (Nguyen et al., 2016; Choi et al., 2023).

Moderate irrigation reduction enhanced root anchorage strength primarily by optimizing root distribution in the 30–60 cm soil layer, thereby contributing secondarily to lodging resistance. Although stem lodging was dominant under TR6S, the root architecture was examined across soil layers to clarify its contribution to lodging rate. Under low water and fertilizer inputs, crop roots generally grow deeper to acquire water and nutrients and alleviate resource stress (Giehl and Von Wirén, 2014). When irrigation water and fertilizer are sufficient, roots tend to remain concentrated in shallow soil layers (Liu et al., 2018). Consistent with this pattern, TRL and TRW in this study decreased continuously in shallow roots, showed an initial increase followed by a decrease in middle-layer roots, and increased continuously in deep roots as water and nitrogen inputs declined (**Figure 6**). Root anchorage strength, which reflects the attachment between roots and soil, is a key indicator of root lodging (Wu et al., 2019). The correlations between root traits (TRL and TRW) and single-plant anchorage strength were the highest in the 30–60 cm middle soil layer, exceeding those in the 0–30 cm and 60–90 cm layers. This indicates that middle-layer root development is critical for lodging resistance under the TR6S pattern in Xinjiang. In addition, TRW contributed more strongly to root anchorage strength than TRL, indicating that root biomass accumulation in the middle soil layer was the core root indicator determining lodging resistance under TR6S. This differs from rain-fed systems, where deep root length often dominates lodging resistance (Berry et al., 2007; Wasson et al., 2012). In arid regions, water availability is the primary factor controlling root spatial distribution and biomass accumulation because soil water potential gradients directly drive root cell elongation, lateral root initiation, and vertical distribution (Cassab et al., 2013; Liu et al., 2018). Nitrogen regulates root systems indirectly, mainly by altering carbon allocation and endogenous hormone balance, and arid conditions further reduce root sensitivity to nitrogen (Kiba and Krapp, 2016; Elazab et al., 2016). Accordingly, the irrigation amount was the dominant factor regulating middle-layer root development in the present study, with a substantially stronger effect than nitrogen application rate (**Figure 6**). This finding supports the regulatory pattern identified above, in which water predominantly regulates roots, whereas nitrogen predominantly regulates stems.

A PLS-SEM model was used to integrate and validate these regulatory pathways. Under the novel TR6S drip-irrigation pattern in Xinjiang, moderate nitrogen reduction enhanced the activities of lignin biosynthesis enzymes (PAL, TAL, 4CL, and CAD), increased lignin content, and improved stalk pole substantial degree. It also strengthened stem mechanical resistance to lodging and ultimately reduced the lodging rate (**Figure 7**). By positively affecting the stalk pole substantial degree and strengthening stem lodging-resistance mechanical properties, the increase in lignin content ultimately reduced the wheat lodging rate, which is consistent with our previous findings. Second, the reduction in the nitrogen application rate decreased the root length and root weight in the 0–30 cm soil layer to a certain extent, while increasing both traits in the 60–90 cm soil layer. This demonstrates that moderate fertilizer reduction is beneficial for promoting deep root penetration. Interestingly, changes in nitrogen application had almost no effect on root length or weight in the 30–60 cm soil layer. When nitrogen was deficient, wheat roots tended to penetrate directly below 60 cm and spread outward to access mineralized nitrogen and residual nitrate nitrogen in deeper soil layers. Under drip irrigation conditions, the nitrogen environment in the 30–60 cm soil layer remained relatively stable because this layer corresponded to a hard soil layer or plow layer, resulting in minimal effects on wheat roots. Compared with nitrogen regulation, changes in irrigation amount more directly influenced root growth status across the 0–90 cm soil profile. Among the different soil layers, the effect of irrigation on roots was greatest in the 0–30 cm shallow soil layer, with an influence coefficient of up to 0.906. This was mainly related to the surface wetting characteristics of drip irrigation (Chen et al., 2015) and the predominant distribution of drip-irrigated wheat roots in the 0–30 cm soil layer (Li et al., 2020). However, compared with shallow-layer roots (0–30 cm), the middle-layer root growth status (30–60 cm) had the greatest effect on root anchorage strength, with an influence coefficient of 0.713. This indicates that in drip-irrigated wheat fields, where root lodging predominates, nitrogen regulation is largely ineffective. Although irrigation regulation was effective, its overall regulatory efficacy was limited because it could not efficiently influence the root status in the 30–60 cm middle soil layer. The limited ability of irrigation to regulate root status in this critical soil layer may be an important reason for the increasing severity and management difficulty of lodging in drip-irrigated wheat in Xinjiang in recent years. Furthermore, XC44 is a representative high-efficiency, high-yielding, and low-stubborn variety. It was selected from over 20 main spring wheat varieties cultivated in Xinjiang through a cluster analysis based on photosynthetic characteristics and lodging resistance (Yang, 2020). The optimal water and nitrogen thresholds and regulatory mechanisms obtained from XC44 may not be directly applicable to all wheat genotypes. However, related research ideas can provide valuable guidance for the study of wheat lodging resistance in other arid and semi-arid regions worldwide.

## 5 Conclusion

Under the novel wide–narrow row TR6S planting pattern, this study confirmed that moderate reductions in water and nitrogen inputs could effectively enhance wheat lodging resistance and maintain a stable grain yield. The corresponding nitrogen application rate was 264.49–283.16 kg·ha^−1^, and the irrigation amount was 4203.55–4237.14 m^3^·ha^−1^. The main regulatory mechanisms are as follows. (1) Moderate nitrogen reduction enhanced the activities of key enzymes involved in lignin biosynthesis in the basal wheat stems, thereby increasing the lignin content and stalk pole degree. This strengthened stem mechanical resistance to lodging and ultimately played a primary role in improving wheat lodging resistance. (2) By optimizing root growth, particularly root weight, in the 30–60 cm soil layer, a moderate reduction in irrigation amount primarily enhanced root anchorage strength and ultimately played a secondary role in improving wheat lodging resistance. (3) Although the interaction between nitrogen application rate and irrigation amount also influenced the regulation of the above indicators, its effect was generally much weaker than the main effects of nitrogen application rate and irrigation amount. (4) We also suggest that in areas using the TR6S planting pattern, water and nitrogen inputs can be moderately reduced through precise management. Long-term high water and nitrogen input levels are unnecessary. Reducing these inputs can further improve wheat lodging resistance and reduce costs. Furthermore, we considered that the lodging type of drip-irrigated wheat might be closely related to the planting pattern. However, regardless of whether root lodging or stem lodging predominates, improving wheat lodging resistance through nitrogen management represents a feasible regulatory strategy, whereas irrigation regulation has a relatively limited effect. This limitation may be attributed to the inability of irrigation to efficiently regulate root status in the critical 30–60 cm soil layer, where root weight is the most important factor for lodging resistance. It may partly explain the increasing severity and management difficulty of lodging in drip-irrigated wheat in Xinjiang in recent years.

## Data Availability

The original contributions presented in the study are included in the article/**Supplementary Material**. Further inquiries can be directed to the corresponding authors.
