# Supplementary material for: Moderate reductions in water and nitrogen inputs enhance lodging resistance of spring wheat under a novel drip irrigation pattern in Xinjiang
Source: Front Plant Sci. 2026 Oct 9;17:1973875. doi: 10.3389/fpls.2026.1973875 (PMC13690664; doi:10.3389/fpls.2026.1973875)
Supplement: Supplementary file 1 [file Supplementaryfile1.docx]

**Supplementary Fig. 1 Schematic diagram of the novel drip irrigation wheat planting pattern TR6S.**

*Notes*: In the TR6S pattern, one drip tube supplies six rows of wheat. Row spacing is 10 cm, lateral spacing between two drip tubes is 80 cm, and border rows of wheat are spaced at 25 cm.

**Supplementary Fig. 2 Schematic diagram of the TR6S seeder.**

**Supplementary Fig. 1**

**Supplementary Fig. 2**
