# Supplementary material for: Moderate reductions in water and nitrogen inputs enhance lodging resistance of spring wheat under a novel drip irrigation pattern in Xinjiang
Source: Front Plant Sci. 2026 Oct 9;17:1973875. doi: 10.3389/fpls.2026.1973875 (PMC13690664; doi:10.3389/fpls.2026.1973875)
Supplement: Supplementary file 2 [file Supplementaryfile2.docx]

Supplementary Table 1. Analysis of variance (ANOVA) and coefficient of variation (C.V) for grain yield and lodging rate of wheat.

| Year | Indicator | Source | df | MS | *F-value* | *P-value* | C.V |
| --- | --- | --- | --- | --- | --- | --- | --- |
| 2024 | Lodging rate | Nitrogen (N) | 2 | 920.042 | 283.666 | <0.001 | 6.579 |
|  |  | Irrigation (W) | 3 | 201.195 | 62.032 | <0.001 |  |
|  |  | N × W | 6 | 47.065 | 14.511 | <0.001 |  |
|  |  | Error | 96 | 3.243 |  |  |  |
|  | Grain yield | Nitrogen (N) | 2 | 34.052 | 302.892 | <0.001 | 4.510 |
|  |  | Irrigation (W) | 3 | 55.027 | 489.471 | <0.001 |  |
|  |  | N × W | 6 | 0.886 | 7.884 | <0.001 |  |
|  |  | Error | 96 | 0.112 |  |  |  |
| 2025 | Lodging rate | Nitrogen (N) | 2 | 634.966 | 246.451 | <0.001 | 6.588 |
|  |  | Irrigation (W) | 3 | 142.585 | 55.342 | <0.001 |  |
|  |  | N × W | 6 | 41.795 | 16.222 | <0.001 |  |
|  |  | Error | 96 | 2.576 |  |  |  |
|  | Grain yield | Nitrogen (N) | 2 | 34.705 | 336.873 | <0.001 | 4.173 |
|  |  | Irrigation (W) | 3 | 47.858 | 464.544 | <0.001 |  |
|  |  | N × W | 6 | 1.827 | 17.731 | <0.001 |  |
|  |  | Error | 96 | 0.103 |  |  |  |

*Notes:* df, degrees of freedom; MS, mean square; N, nitrogen application rate; W, irrigation amount; N×W, interaction between nitrogen application rate and irrigation amount. *P-value* < 0.001 indicate significant differences at the 0.001 probability level. Coefficient of variation (C.V) was calculated as the square root of error mean square (MSE) divided by the grand mean of the corresponding parameter, multiplied by 100.

Supplementary Table 2. Analysis of variance (ANOVA) and coefficient of variation (C.V) for mechanical indicators related to wheat lodging resistance.

| Year | Indicator | Source | df | MS | *F-value* | *P-value* | C.V |
| --- | --- | --- | --- | --- | --- | --- | --- |
| 2024 | SBS | Nitrogen (N) | 2 | 9.447 | 27.509 | <0.001 | 8.546 |
|  |  | Irrigation (W) | 3 | 2.811 | 8.185 | <0.001 |  |
|  |  | N × W | 6 | 1.043 | 3.036 | 0.024 |  |
|  |  | Error | 348 | 0.343 |  |  |  |
|  | SRPS | Nitrogen (N) | 2 | 54.954 | 20.021 | <0.001 | 7.790 |
|  |  | Irrigation (W) | 3 | 18.979 | 6.915 | 0.002 |  |
|  |  | N × W | 6 | 3.233 | 1.178 | 0.362 |  |
|  |  | Error | 348 | 2.745 |  |  |  |
|  | SPAS | Nitrogen (N) | 2 | 8.945 | 4.850 | 0.001 | 6.003 |
|  |  | Irrigation (W) | 3 | 84.142 | 45.622 | <0.001 |  |
|  |  | N × W | 6 | 2.311 | 1.253 | 0.377 |  |
|  |  | Error | 96 | 1.844 |  |  |  |
| 2025 | SBS | Nitrogen (N) | 2 | 19.626 | 62.072 | <0.001 | 8.179 |
|  |  | Irrigation (W) | 3 | 7.246 | 22.917 | <0.001 |  |
|  |  | N × W | 6 | 2.029 | 6.419 | <0.001 |  |
|  |  | Error | 348 | 0.316 |  |  |  |
|  | SRPS | Nitrogen (N) | 2 | 50.118 | 25.806 | <0.001 | 6.446 |
|  |  | Irrigation (W) | 3 | 16.596 | 8.546 | <0.001 |  |
|  |  | N × W | 6 | 2.237 | 1.152 | 0.364 |  |
|  |  | Error | 348 | 1.942 |  |  |  |
|  | SPAS | Nitrogen (N) | 2 | 10.097 | 4.530 | 0.001 | 6.670 |
|  |  | Irrigation (W) | 3 | 156.005 | 69.990 | <0.001 |  |
|  |  | N × W | 6 | 2.652 | 1.190 | 0.635 |  |
|  |  | Error | 96 | 2.229 |  |  |  |

*Notes:* SBS, SRPS and SPAS represent stalk bending strength, stalk rind penetration strength and single-plant anchorage strength, respectively. df, degrees of freedom; MS, mean square; N, nitrogen application rate; W, irrigation amount; N×W, interaction between nitrogen application rate and irrigation amount. *P-value* < 0.001 indicate significant differences at the 0.001 probability level. Coefficient of variation (C.V) was calculated as the square root of error mean square (MSE) divided by the grand mean of the corresponding parameter, multiplied by 100.

Supplementary Table 3. Analysis of variance (ANOVA) and coefficient of variation (C.V) for stalk pole substantial degree and lignin content of wheat.

| Year | Indicator | Source | df | MS | *F-value* | *P-value* | C.V |
| --- | --- | --- | --- | --- | --- | --- | --- |
| 2024 | SPSD | Nitrogen (N) | 2 | 0.168 | 9.280 | <0.001 | 9.882 |
|  |  | Irrigation (W) | 3 | 0.066 | 3.620 | 0.044 |  |
|  |  | N × W | 6 | 0.050 | 2.740 | 0.046 |  |
|  |  | Error | 348 | 0.018 |  |  |  |
|  | SLC | Nitrogen (N) | 2 | 6317.461 | 149.793 | <0.001 | 2.084 |
|  |  | Irrigation (W) | 3 | 3080.370 | 73.038 | <0.001 |  |
|  |  | N × W | 6 | 390.214 | 9.252 | <0.001 |  |
|  |  | Error | 96 | 42.175 |  |  |  |
| 2025 | SPSD | Nitrogen (N) | 2 | 0.299 | 20.734 | <0.001 | 9.053 |
|  |  | Irrigation (W) | 3 | 0.109 | 7.541 | 0.001 |  |
|  |  | N × W | 6 | 0.042 | 2.918 | 0.028 |  |
|  |  | Error | 348 | 0.014 |  |  |  |
|  | SLC | Nitrogen (N) | 2 | 7787.927 | 193.955 | <0.001 | 2.041 |
|  |  | Irrigation (W) | 3 | 3748.208 | 93.348 | <0.001 |  |
|  |  | N × W | 6 | 522.417 | 13.011 | <0.001 |  |
|  |  | Error | 96 | 40.153 |  |  |  |

*Notes:* SPSD and SLC represent stalk pole substantial degree and stalk lignin content, respectively. df, degrees of freedom; MS, mean square; N, nitrogen application rate; W, irrigation amount; N×W, interaction between nitrogen application rate and irrigation amount. *P-value* < 0.001 indicate significant differences at the 0.001 probability level. Coefficient of variation (C.V) was calculated as the square root of error mean square (MSE) divided by the grand mean of the corresponding parameter, multiplied by 100.

Supplementary Table 4. Analysis of variance (ANOVA) and coefficient of variation (C.V) for key lignin biosynthetic enzymes of wheat stalk.

| Year | Indicator | Source | df | MS | *F-value* | *P-value* | C.V |
| --- | --- | --- | --- | --- | --- | --- | --- |
| 2024 | PAL | Nitrogen (N) | 2 | 2.875 | 183.703 | <0.001 | 2.803 |
|  |  | Irrigation (W) | 3 | 1.738 | 111.028 | <0.001 |  |
|  |  | N × W | 6 | 0.173 | 11.030 | <0.001 |  |
|  |  | Error | 96 | 0.016 |  |  |  |
|  | TAL | Nitrogen (N) | 2 | 0.281 | 29.131 | <0.001 | 12.832 |
|  |  | Irrigation (W) | 3 | 0.195 | 20.164 | <0.001 |  |
|  |  | N × W | 6 | 0.027 | 2.810 | 0.271 |  |
|  |  | Error | 96 | 0.010 |  |  |  |
|  | 4CL | Nitrogen (N) | 2 | 1.252 | 54.008 | <0.001 | 12.913 |
|  |  | Irrigation (W) | 3 | 0.467 | 20.145 | <0.001 |  |
|  |  | N × W | 6 | 0.123 | 5.311 | 0.001 |  |
|  |  | Error | 96 | 0.023 |  |  |  |
|  | CAD | Nitrogen (N) | 2 | 0.138 | 59.517 | <0.001 | 11.694 |
|  |  | Irrigation (W) | 3 | 0.034 | 14.737 | <0.001 |  |
|  |  | N × W | 6 | 0.002 | 0.948 | 0.480 |  |
|  |  | Error | 96 | 0.002 |  |  |  |
| 2025 | PAL | Nitrogen (N) | 2 | 1.074 | 54.997 | <0.001 | 3.144 |
|  |  | Irrigation (W) | 3 | 0.607 | 31.112 | <0.001 |  |
|  |  | N × W | 6 | 0.080 | 4.076 | 0.006 |  |
|  |  | Error | 96 | 0.020 |  |  |  |
|  | TAL | Nitrogen (N) | 2 | 0.932 | 96.049 | <0.001 | 13.777 |
|  |  | Irrigation (W) | 3 | 0.553 | 56.969 | <0.001 |  |
|  |  | N × W | 6 | 0.060 | 6.168 | <0.001 |  |
|  |  | Error | 96 | 0.010 |  |  |  |
|  | 4CL | Nitrogen (N) | 2 | 1.266 | 58.784 | <0.001 | 12.329 |
|  |  | Irrigation (W) | 3 | 0.439 | 20.364 | <0.001 |  |
|  |  | N × W | 6 | 0.087 | 4.060 | 0.001 |  |
|  |  | Error | 96 | 0.022 |  |  |  |
|  | CAD | Nitrogen (N) | 2 | 0.159 | 53.189 | <0.001 | 13.237 |
|  |  | Irrigation (W) | 3 | 0.085 | 28.323 | <0.001 |  |
|  |  | N × W | 6 | 0.002 | 0.585 | 0.739 |  |
|  |  | Error | 96 | 0.003 |  |  |  |

*Notes:* PAL, TAL, 4CL and CAD represent phenylalanine ammonia-lyase, tyrosine ammonia-lyase, 4-Coumarate-CoA ligase and cinnamyl alcohol dehydrogenase, respectively. df, degrees of freedom; MS, mean square; N, nitrogen application rate; W, irrigation amount; N×W, interaction between nitrogen application rate and irrigation amount. *P-value* < 0.001 indicate significant differences at the 0.001 probability level. Coefficient of variation (C.V) was calculated as the square root of error mean square (MSE) divided by the grand mean of the corresponding parameter, multiplied by 100.

Supplementary Table 5. Analysis of variance (ANOVA) and coefficient of variation (C.V) for total root weight and total root length of wheat in different soil layers.

| Year | Indicator | Source | df | MS | *F-value* | *P-value* | C.V |
| --- | --- | --- | --- | --- | --- | --- | --- |
| 2024 | TRW of 0-30 cm | Nitrogen (N) | 2 | 18.041 | 9.809 | <0.001 | 9.065 |
|  |  | Irrigation (W) | 3 | 150.157 | 81.637 | <0.001 |  |
|  |  | N × W | 6 | 0.966 | 0.525 | 0.783 |  |
|  |  | Error | 96 | 1.839 |  |  |  |
|  | TRW of 30-60 cm | Nitrogen (N) | 2 | 1.606 | 7.332 | 0.042 | 7.971 |
|  |  | Irrigation (W) | 3 | 29.081 | 132.738 | <0.001 |  |
|  |  | N × W | 6 | 0.981 | 4.477 | 0.004 |  |
|  |  | Error | 96 | 0.219 |  |  |  |
|  | TRW of 60-90 cm | Nitrogen (N) | 2 | 0.316 | 6.410 | <0.001 | 13.353 |
|  |  | Irrigation (W) | 3 | 3.633 | 73.740 | <0.001 |  |
|  |  | N × W | 6 | 0.033 | 0.660 | 0.783 |  |
|  |  | Error | 96 | 0.049 |  |  |  |
|  | TRL of 0-30 cm | Nitrogen (N) | 2 | 20.250 | 6.987 | <0.001 | 10.141 |
|  |  | Irrigation (W) | 3 | 205.425 | 70.877 | <0.001 |  |
|  |  | N × W | 6 | 2.168 | 0.748 | 0.634 |  |
|  |  | Error | 96 | 2.898 |  |  |  |
|  | TRL of 30-60 cm | Nitrogen (N) | 2 | 1.428 | 4.420 | 0.028 | 7.146 |
|  |  | Irrigation (W) | 3 | 10.180 | 31.513 | <0.001 |  |
|  |  | N × W | 6 | 2.196 | 6.799 | <.001 |  |
|  |  | Error | 96 | 0.323 |  |  |  |
|  | TRL of 60-90 cm | Nitrogen (N) | 2 | 1.158 | 4.210 | 0.025 | 12.980 |
|  |  | Irrigation (W) | 3 | 3.695 | 13.437 | <0.001 |  |
|  |  | N × W | 6 | 0.080 | 0.291 | 0.936 |  |
|  |  | Error | 96 | 0.275 |  |  |  |
| 2025 | TRW of 0-30 cm | Nitrogen (N) | 2 | 19.267 | 11.396 | <0.001 | 9.278 |
|  |  | Irrigation (W) | 3 | 107.870 | 63.806 | <0.001 |  |
|  |  | N × W | 6 | 1.878 | 1.111 | 0.385 |  |
|  |  | Error | 96 | 1.691 |  |  |  |
|  | TRW of 30-60 cm | Nitrogen (N) | 2 | 2.286 | 6.510 | 0.042 | 9.835 |
|  |  | Irrigation (W) | 3 | 23.107 | 65.790 | <0.001 |  |
|  |  | N × W | 6 | 1.068 | 3.040 | 0.048 |  |
|  |  | Error | 96 | 0.351 |  |  |  |
|  | TRW of 60-90 cm | Nitrogen (N) | 2 | 0.254 | 7.952 | <0.001 | 11.542 |
|  |  | Irrigation (W) | 3 | 1.893 | 59.218 | <0.001 |  |
|  |  | N × W | 6 | 0.037 | 1.161 | 0.332 |  |
|  |  | Error | 96 | 0.032 |  |  |  |
|  | TRL of 0-30 cm | Nitrogen (N) | 2 | 37.281 | 12.763 | <0.001 | 9.835 |
|  |  | Irrigation (W) | 3 | 200.101 | 68.506 | <0.001 |  |
|  |  | N × W | 6 | 3.330 | 1.140 | 0.078 |  |
|  |  | Error | 96 | 2.921 |  |  |  |
|  | TRL of 30-60 cm | Nitrogen (N) | 2 | 1.637 | 5.510 | 0.022 | 6.976 |
|  |  | Irrigation (W) | 3 | 5.639 | 18.978 | <0.001 |  |
|  |  | N × W | 6 | 2.264 | 7.620 | <0.001 |  |
|  |  | Error | 96 | 0.297 |  |  |  |
|  | TRL of 60-90 cm | Nitrogen (N) | 2 | 1.180 | 4.680 | 0.021 | 13.121 |
|  |  | Irrigation (W) | 3 | 5.850 | 23.199 | <0.001 |  |
|  |  | N × W | 6 | 0.130 | 0.517 | 0.790 |  |
|  |  | Error | 96 | 0.252 |  |  |  |

*Notes:* TRW and TRL represent total root weight and total root length , respectively. df, degrees of freedom; MS, mean square; N, nitrogen application rate; W, irrigation amount; N×W, interaction between nitrogen application rate and irrigation amount. *P-value* < 0.001 indicate significant differences at the 0.001 probability level. Coefficient of variation (C.V) was calculated as the square root of error mean square (MSE) divided by the grand mean of the corresponding parameter, multiplied by 100.
